# A Bioinspired Artificial Injury Response System Based on a Robust Polymer Memristor to Mimic a Sense of Pain, Sign of Injury, and Healing

**DOI:** 10.1002/advs.202200629

**Published:** 2022-03-25

**Authors:** Xiaojie Xu, En Ju Cho, Logan Bekker, A. Alec Talin, Elaine Lee, Andrew J. Pascall, Marcus A. Worsley, Jenny Zhou, Caitlyn C. Cook, Joshua D. Kuntz, Seongkoo Cho, Christine A. Orme

**Affiliations:** ^1^ Lawrence Livermore National Laboratory 7000 East Avenue Livermore CA 94550 USA; ^2^ Sandia National Laboratories Livermore CA 94551 USA

**Keywords:** artificial nociceptor, electronic skin, FK‐800, flexible memristor, memristor

## Abstract

Flexible electronic skin with features that include sensing, processing, and responding to stimuli have transformed human–robot interactions. However, more advanced capabilities, such as human‐like self‐protection modalities with a sense of pain, sign of injury, and healing, are more challenging. Herein, a novel, flexible, and robust diffusive memristor based on a copolymer of chlorotrifluoroethylene and vinylidene fluoride (FK‐800) as an artificial nociceptor (pain sensor) is reported. Devices composed of Ag/FK‐800/Pt have outstanding switching endurance >10^6^ cycles, orders of magnitude higher than any other two‐terminal polymer/organic memristors in literature (typically 10^2^–10^3^ cycles). In situ conductive atomic force microscopy is employed to dynamically switch individual filaments, which demonstrates that conductive filaments correlate with polymer grain boundaries and FK‐800 has superior morphological stability under repeated switching cycles. It is hypothesized that the high thermal stability and high elasticity of FK‐800 contribute to the stability under local Joule heating associated with electrical switching. To mimic biological nociceptors, four signature nociceptive characteristics are demonstrated: threshold triggering, no adaptation, relaxation, and sensitization. Lastly, by integrating a triboelectric generator (artificial mechanoreceptor), memristor (artificial nociceptor), and light emitting diode (artificial bruise), the first bioinspired injury response system capable of sensing pain, showing signs of injury, and healing, is demonstrated.

## Graphical abstract

A flexible polymeric memristor with cycling endurance matching inorganic memristors is demonstrated to have the key properties of an artificial nociceptor. In‐situ conductive atomic force microscopy is used to dynamically switch individual filaments to provide insights on the switching endurance. The device is integrated into a bioinspired injury response system capable of sensing pain, showing signs of injury, and healing.

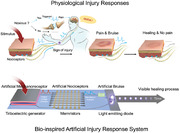

## 1 Introduction

In the past two decades, humanoid robotics has rapidly advanced with the aim of revolutionizing the way machines interact with humans and the environment.^[^ ^1^, ^2^, ^3^, ^4^ ^]^ Humanoid robot design seeks to use electronic systems to reproduce key human characteristics, including sensing, processing, and responding to environmental stimuli.^[^ ^5^, ^6^ ^]^ Inspired by the human skin—one of the principle organs receiving sensory input from the physical world—electronic skin has been developed for robots with various bioinspired features, such as a sense of touch enabled by arrays of piezoelectric/triboelectric generators,^[^ ^7^, ^8^, ^9^ ^]^ a sense of heat by temperature sensors,^[^ ^10^, ^11^, ^12^ ^]^ and a sense of environmental pollutants by chemical and gas sensors.^[^ ^13^, ^14^, ^15^ ^]^ However, more advanced physiological functions, such as human‐like injury response remain less developed. For example, when our bodies experience noxious stimuli from the external environment, a series of complex physiological responses are triggered to protect us from harm or further damage, including a sense of pain, visible signs of injury, and healing. To the best of our knowledge, this advanced human‐like physiological protection modality has not yet been demonstrated in electronic skins.

Pain, defined as a “complex constellation of unpleasant sensory, emotional and cognitive experiences provoked by real or perceived tissue damage and manifested by certain autonomic, psychological, and behavioral reactions,” is a protective somatic modality.^[^ ^16^ ^]^ The detection of physical pain is through nociceptors, a type of sensory receptor that encodes the noxious stimuli into neural signals, and sends “possible threat” signals to the spinal cord and the brain, triggering a variety of physiological and behavioral responses.^[^ ^17^ ^]^ Lacking the ability to feel physical pain, known as congenital insensitivity to pain, is an extremely dangerous condition as it often leads to unrealized infections, self‐mutilation, and shortened life spans.^[^ ^18^, ^19^ ^]^

A biological nociceptor has four characteristic features: i) a threshold pain level to initiate triggering, ii) continuous triggering in response to repeating pain signals, known as “no adaptation,” iii) relaxation after the noxious stimuli has stopped, and iv) sensitization, or lower thresholds while injured.^[^ ^16^ ^]^ Mimicking this complex set of responses typically requires complicated multicomponent circuits when using conventional complementary metal oxide semiconductor devices.^[^ ^20^, ^21^ ^]^ However, it has been demonstrated that a two terminal memristor can simulate nociceptive behaviors.^[^ ^22^, ^23^, ^24^, ^25^, ^26^ ^]^ A memristor is a memory resistor that can switch and retain internal resistance states according to their history of applied voltage.^[^ ^27^, ^28^ ^]^ Although simple in principle, a memristor can have rich switching dynamics that lead to multistate characteristics, history‐dependent resistance and electrical stimuli‐dependent thresholds.^[^ ^27^, ^29^, ^30^, ^31^ ^]^ These functions share interesting similarities with those of biosensory systems (synaptic and neuronal signals), making the memristor a promising candidate as an artificial synapse and neuron.^[^ ^27^, ^32^ ^]^ The vast majority of memristors investigated to date are nonvolatile, and are being actively investigated as synaptic elements for in‐memory computing applications.^[^ ^32^, ^33^, ^34^ ^]^ By contrast, diffusive memristors only retain state when potential is applied. The volatile threshold switching with unique temporal evolution dynamics is particularly interesting to emulate synaptic plasticity with relaxation process and have been successfully demonstrated as artificial neuronal elements as well as access devices.^[^ ^30^ ^]^

The memristive switching behavior is mainly attributed to the formation and rupture of local conductive filaments associated with ion and/or vacancy motion in electric fields.^[^ ^35^, ^36^ ^]^ Inorganic metal oxides and organic/polymer thin materials (with a thickness of tens to hundreds of nanometers) have been extensively investigated as insulating layers.^[^ ^37^, ^38^ ^]^ The mobile ion species can be oxygen vacancies in the oxides,^[^ ^39^ ^]^ or metal ions originating from the active electrodes (such as Ag or Cu).^[^ ^40^ ^]^ Once the conductive filaments bridge the two electrodes, the devices switch from insulating to conductive, with “on” current densities that can exceed 10^6^ A cm^−2^.^[^ ^37^ ^]^ Due to the specific nature of organic materials, including mechanical flexibility, conformability, facile, and low‐cost preparation methods and rich switching mechanisms, considerable effort and progress has been made in the past two decades in developments of organic memristors.^[^ ^26^, ^41^, ^42^ ^]^ A variety of organic materials and neuromorphic device configurations have thus been proposed in organic memristors for bio‐related applications.^[^ ^25^, ^43^, ^44^ ^]^ However, the high “on” current density will potentially pose a key challenge for organic/polymer memristors: namely poor switching endurance (typically 10–10^3^ cycles) attributed to local Joule heating and insufficient heat dissipation.^[^ ^37^, ^38^ ^]^Although, inorganic memristors are more robust with cycle numbers >10^6^, they are less desirable for wearable applications due to their inherent brittleness and fabrication methods that are not easily adapted to complex shapes, such as wires.^[^ ^37^, ^45^ ^]^

To overcome one of the key challenges for organic memristors (poor endurance) and reliably emulate nociceptive behaviors, we design a new, flexible, and robust diffusive memristor based on a copolymer composed of chlorotrifluoroethylene (CTFE) and vinylidene fluoride (VF_2_), known as FK‐800. FK‐800 is selected due to its high degradation temperature, low thermal expansion coefficient, and low elastic modulus,^[^ ^46^, ^47^ ^]^ all of which is expected to help maintain structural stability under local heat loads associated with electric switching. Silver (Ag) electrode is used as the active diffusion source, and platinum (Pt) as the counter electrode. Our flexible memristors made of Ag/FK‐800/Pt display outstanding switching endurance of >10^6 ^cycles, which is orders of magnitude higher than almost all other two terminal polymer/organic memristors reported in literature^[^ ^48^, ^49^, ^50^, ^51^, ^52^, ^53^, ^54^, ^55^ ^]^ (see Table S1, Supporting Information) and comparable to that of the state‐of‐art inorganic memristors.^[^ ^37^, ^38^ ^]^ In situ conductive atomic force microscopy is employed to study the superior endurance of FK‐800, and it shows an outstanding morphological stability under electric switching compared with conventional polymers, such as polyvinylidene fluoride–polyvinylidene fluoride (PVDF), which is likely attributed to its high thermal stability and mechanical elasticity.Moreover, with the successful realization of a flexible robust diffusive memristor that exhibits nociceptive behaviors, we demonstrate the first bioinspired injury response system for artificial skin which includes a sense of pain, signs of injury, and the healing process in response to noxious stimuli. This artificial self‐protection modality is realized by the effective integration of an artificial mechanoreceptor (triboelectric generator), an artificial nociceptor (memristor), and an artificial bruise (light emitting diode).

## 2 A Flexible Robust Nociceptive Memristor Based on FK‐800

**Figure 1:**
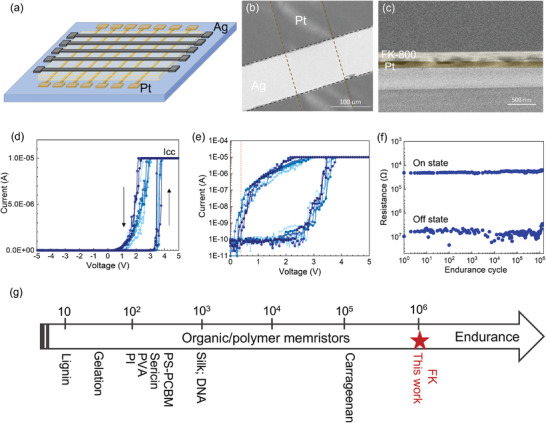
Characteristics of FK‐800 based memristors. a) Schematic illustration of the FK‐800 memristor cross‐bar arrays with the structure of Pt/FK‐800/Ag. b) Plan view SEM image of the FK‐800 memristors with a junction size of ≈100 µm × 100 µm. c) Cross‐sectional SEM image of the FK‐800 memristor; the thickness of FK‐800 film is ≈200 nm. d) *I*–*V* cycles (0 →5 V → −5 V → 0) of an FK‐800 memristor at a sweep rate of 0.3 V s^−1^. e) The corresponding *I*–*V* sweeps on a log scale. f) Endurance test of the FK‐800 memristor with 10^6^ cycles that switch between the “on” and “off” states; the testing on and off voltage pulses applied were 4.5 and 0.2 V with a pulse width of 0.5 ms pulse and pulse interval of 2 ms. g) Comparison of the endurance of the high‐performance organic/polymer memristors reported in literature.^[^ ^41^, ^42^, ^43^, ^44^, ^45^, ^46^, ^47^, ^48^ ^]^

Memristor devices were fabricated in cross‐bar arrays (**Figure** **1**a) with silver electrodes serving as the source of mobile ions under an oxidizing potential and platinum serving as the counter electrode. A ≈200 nm thick film of FK‐800 separates the electrodes creating a 100 µm × 100 µm stacked structure of Ag/FK‐800/Pt at each junction (Figure 1b,c). The root mean square (RMS) roughness of FK‐800 device films as a function of area size is shown in Figure S1 of the Supporting Information. Our devices have an RMS roughness of 4.2 ± 0.5 nm over a 1 *μ*m^2^ area that results from the crystalline grains and grain boundaries. At the device scale, our RMS roughness is <4% of the thickness. Although outside the scope of this paper, we note that the grain size and RMS roughness are functions of our annealing temperature profile and can be systematically tuned. FK‐800 is a random copolymer nominally composed of 75% CTFE and 25% vinylidene fluoride (VF_2_). FK‐800 was chosen for its superior thermal stability (thermal degradation temperature of 400 °C), lower thermal expansion coefficient (≈55 ± 10 μmm^−1^ K^−1^ at 293 K) and higher mechanical elasticity (Young's modulus of 0.16 GPa).^[^ ^46^, ^47^ ^]^ See Table S2 of the Supporting Information for a comparison of the physical properties of polymers that have been explored in memristors. Most testing is performed on devices deposited on silicon substrates. However, to test performance during bending, flexible PET substrates are used.

We first measure the threshold switching behavior and robustness of the devices during cyclic current–voltage (*I*–*V*) sweeps at a sweep rate of ≈0.3 V s^−1^ (Figure 1d,e). During the forward sweep (0→5 V), the device rapidly switches from an off‐state to an on‐state where the current rises quickly to the compliance (*I*_cc_ of 10 µA) at a threshold voltage of *V*_on_ = 3.40 ± 0.06 V. During the reverse sweep (5 V→ −5 V) the current decreases at voltages less than 2 V and returns to the off‐state at bias voltages ≤0.2 V. The first *I*–*V* sweep and subsequent sweeps in Figure 1d overlap with only a slight offset, suggesting an electroforming‐free switching behavior and low cycle‐to‐cycle variability. Due to the dynamic nature of the switching process, the threshold voltage is not fixed, but can be affected by the characteristics of the bias, such as sweep rates.^[^ ^56^ ^]^ A faster sweep rate results in a higher threshold voltage (see details in Figure S2, Supporting Information). To study the device‐to‐device variability, ten additional FK‐800 memristors are fabricated and tested under the same conditions (Figure S3, Supporting Information). The average threshold voltage of these devices is 3.40 ± 0.08 V, and the small relative standard deviation (≈2.3%) indicates a low device‐to‐device variability. The response speed (set time at 5 V, see details in Figure S4, Supporting Information) of the devices is ≈340 µs, which is faster than that of biological synapses (≈10–100 ms). To demonstrate the mechanical flexibility of the FK‐800 memristors, similar devices are also fabricated on PET substrates and tested under flat and bent conditions using a bending radius of ≈8 mm (see details in Figure S5, Supporting Information). Negligible differences between the flat and bent switching thresholds are found, suggesting that FK‐800 memristors are promising for wearable applications.

To study the cycling endurance of the devices, pulse cycles consisting of alternating *V*_on_ of 4.5 V and *V*_off_ of 0.2 V with a pulse width of 500 µs and pulse interval of 2 ms are used to switch the device between conducting and insulating states. We observe no noticeable degradation following 10^6^ ON/OFF cycles (Figure 1f; Figure S6, Supporting Information). To the best of our knowledge, the endurance cycling of our devices is the highest reported to date for two‐terminal organic/polymeric memristors, which are typically in the range of 10^2^–10^3^ cycles (see Table S1, Supporting Information).^[^ ^48^, ^49^, ^50^, ^51^, ^52^, ^53^, ^54^, ^55^ ^]^ Note that higher cycle stability has only been observed for three‐terminal electrochemical devices that do not form conductive filaments.^[^ ^57^ ^]^ Notably, the outstanding switching robustness of the FK‐800 memristors is comparable to the state‐of‐art inorganic memristors.^[^ ^28^, ^37^ ^]^

## 3 Conductive Atomic Force Microscopy (AFM) to Correlate Filament Formation with Morphology

To shed light on the underlying reasons for high cycling endurance of FK‐800 memristors, we compare their performance to control devices made of PVDF polymer thin films, with the same device geometry and comparable thickness. Similar to most other polymer memristors reported, the PVDF device only sustains on‐off switching for ≈150 cycles before permanent breakdown (Figure S7, Supporting Information). Literature reports suggest that local Joule heating caused by current flowing through the filaments plays an important role in permanently shunting devices.^[^ ^37^, ^58^ ^]^ Here we propose that filament formation and heating can cause irreversible morphological deformation of the polymer film, which reduces the switching endurance of organic/polymer memristors. To verify our hypothesis, conductive atomic force microscopy (c‐AFM) was employed to measure the *I*–*V* response of single filaments and to correlate filament formation with polymer film morphology before and after switching.

**Figure 2:**
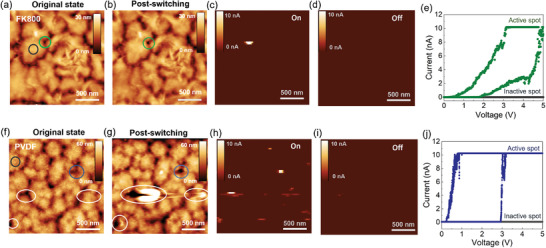
Comparing the morphological integrity of FK‐800 and PVDF‐based memristors before and after switching via conductive AFM. Topography image of the FK‐800 a) before applying a voltage and b) after cycling. c) Corresponding current map in the on‐state using a bias of 5 V and a compliance current of 10 nA. d) Corresponding current map in the off‐state using a bias of 0.2 V. e) *I*–*V* sweep at the active spot indicated in the green circle in (a) and inactive spot indicated by the grey circle in (a). There are no obvious changes in morphology due to switching. To compare, a PVDF device was scanned under the same conditions. Topography image of the PVDF film f) before applying a voltage and g) after cycling. h) Corresponding current map in the on‐state using a bias of 5 V and a compliance current of 10 nA. i) Corresponding current map in the off‐state using a bias of 0.2 V. j) *I*–*V* sweep at the active spot indicated in the blue circle in (f) and inactive spot indicated in the gray circle in (f). White circles in (f) and (g) highlight active spots where there is evidence of irreversible morphological changes due to switching. All images are 2 µm × 2 µm. The vertical scale is provided as an inset.

Topographic maps of as‐synthesized FK‐800 (**Figure** **2**a) and PVDF (Figure 2f) films deposited on Ag‐coated Si substrates show continuous films composed of ≈0.5 µm diameter crystalline grains (bright regions) separated by their grain boundaries (dark regions). Film continuity between grains is verified by phase contrast images in Figure S8 of the Supporting Information. To determine the location of filament formation and to investigate the switching robustness of these films, c‐AFM is used to measure current maps during multiple “on” and “off” cycles. In this set up, the c‐AFM tip plays the role of the counter electrode. A bias voltage is applied between the Ag layer and a Pt‐coated AFM tip generating a local electric field across the polymer film (see the detailed c‐AFM setup in Figure S9, Supporting Information). Current maps of an FK‐800 sample with an on‐bias of 5 V (Figure 2c) show that current is localized to one position within the 2 µm × 2 µm field of view, as indicated by the single bright spot. This spot, which occurs at a grain boundary where the polymer is thinner, is shown as a green circle in the topographic image. When the bias voltage is switched to an off‐voltage, 0.2 V, the active region is turned off (Figure 2d). These data illustrates dynamic formation and rupture of an individual filament that forms between the silver substrate and the Pt AFM tip.

The process for filament formation has been discussed extensively in the literature.^[^ ^30^, ^36^ ^]^ When an on‐bias of 5 V is applied across the thin film (≈30 nm), the metal atoms at the positive Ag electrode interface become oxidized (Ag → Ag^+^ + e^−^). The silver ions then drift across the polymer gap to the platinum electrode due to the large electric field where they are then reduced back to metallic silver.^[^ ^36^, ^40^ ^]^ Ag deposits observed on the Pt tip after cycling (Figure S9, Supporting Information) support this picture. Due to the localized flux of Ag ions, Ag islands that nucleate and grow on the Pt tip form filaments that eventually bridge the electrodes, lowering the resistance and allowing a local current to flow. When the bias is reduced or removed, the filament ruptures and the active region reset to its insulating state. This rearrangement of Ag atoms when the ion flux is removed, suggests that the high‐aspect ratio Ag filaments are kinetically rather than thermodynamically stable in this matrix material. When the voltage is removed, Ag atoms redistribute and the filament relaxes to reduce its surface to volume ratio.^[^ ^30^, ^59^ ^]^

Current maps after multiple subsequent on–off cycles show that the FK‐800 film can be consistently turned on and off. The conductive filament occurred at almost the same spot every cycle (Figure S10, Supporting Information), which demonstrates reliable switching at preferential sites. *I*–*V* sweeps are compared between the active region and a nonactive region. As seen in Figure 2e, the active spot shows a threshold switching behavior while the nonactive region remains insulating. The conductive filaments are more likely to form at grain boundaries, which is likely due to the lower energy barrier associated with thinner areas and looser packing of the polymer chains in the grain boundary regions. Additionally, the electric fields may be concentrated at the boundary due to a lower dielectric constant compared to the surrounding (denser) polymer. The morphology of the FK‐800 film after switching cycles is tracked and shown in Figure 2b and Figure S11 (Supporting Information). Negligible variations are found between the initial state and postswitching state, which supports our assertion that FK‐800 is morphologically stable under switching conditions.

By contrast, we observe significant morphological changes to the PVDF film after electrical switching, as indicated by the white circles in Figure 2f,g. First, multiple spots are activated when scanning with a bias of 5 V. As is found for the FK‐800 films, filaments preferentially occur at grain boundaries. However, the distribution of the conductive filaments is more stochastic and differed for every cycle (Figure S10, Supporting Information). Second, noticeable morphological variations are observed after 3 cycles of switching. Specifically, the polymer chains in the activated regions are depleted by 2–3 times in thickness and merged into the neighboring grains (Figure S12, Supporting Information). These observations support our hypothesis that irreversible morphological changes play an important role in the poor endurance of conventional polymer memristors, such as PVDF.

Overall this study suggests that FK‐800 grain boundaries play an important role in filament formation by providing preferential locations that reduce stochasticity. Although outside the scope of this paper, we note that grain‐boundary engineering may provide a means to improve performance and scale device size. The grain size and RMS roughness of FK‐800 films are functions of our annealing temperature profile. Topographic maps of our device films (Figure S1, Supporting Information) shows that we have uniform distribution of grains and grain boundaries in the FK‐800 film when zoomed out to the scale of our device (100 µm × 100 µm); however, it may be desirable to have smaller grain sizes for smaller device geometries. Given that conductive filaments are more likely to form at the grain boundaries, optimizing the grain sizes of FK‐800 films to guide/confine the filament formation may be a potential route to further reduce the cycle‐to‐cycle and device‐to‐device variability. We note that smaller grain sizes also reduce the height variation and correspondingly the RMS roughness.

Moreover, this study illustrates that FK‐800 does not deform during switching whereas PVDF does, which we attribute to its thermo‐mechanical properties. When the conductive filaments are formed, local Joule heating associated with high current density will pose a challenge for switching endurance, particularly for polymers as they generally suffer from a relatively low thermal stability and high thermal expansion coefficient. However, FK‐800 based memristors exhibit outstanding endurance. Compared with most conventional polymers, FK‐800 has a higher thermal stability, a lower thermal expansion coefficient and a higher elasticity (see Table S2, Supporting Information), which is expected to favor higher structural stability associated with electrical switching and verified by the negligible morphology change before and after multiple switching cycles.

## 4 An Artificial Nociceptor Mimicked by an FK‐800 Memristor with the Four Signature Nociceptive Behaviors

**Figure 3:**
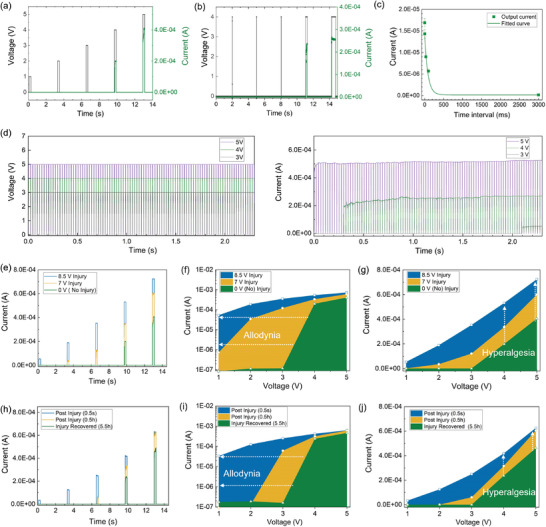
An artificial nociceptor based on an FK‐800 memristor with four signature nociceptive characteristics: threshold, no adaptation, relaxation, and sensitization. a) Pulse level measurement: a train of 200 ms wide voltage pulses (black) with different amplitudes (1, 2, 3, 4, and 5 V) and the corresponding output currents (green). The interval time between pulses was with 3 s. b) Pulse width measurement: a train pulse of 4 V (black) with different pulse width (0.5, 1, 10, 200, and 500 ms) and the corresponding output currents (green). The interval time between pulses was with 3 s. c) Relaxation: the current response at 2 V (200 ms) after a train pulse of 4 V (200 ms) with different interval time: 0.1, 1, 30, 100, and 3000 ms. d) Current response of the device to 100 voltage pulses (pulse width of 20 ms and pulse interval of 2 ms) with different amplitude (3, 4, and 5 V). The higher the pulse level, the shorter the incubation time. e–j) Demonstration of the allodynia and hyperalgesia features. (e) The output currents at 200 ms voltage pulses with different amplitudes (1, 2, 3, 4, and 5 V) 3 min after different injury pulses (8.5 V – blue, 7 V – yellow, 0 V – green/no injury). The corresponding maximum output currents at different pulse amplitudes in (f) log scale and (g) linear scale. (h) The output currents at 200 ms voltage pulses with different amplitudes (1, 2, 3, 4, and 5 V) after the injury pulse of 7 V (200 ms) with different time intervals, 500 ms (blue), 30 min (yellow), and 5.5 h (green). The corresponding maximum output currents at different pulse amplitudes in (i) log scale and (j) linear scale. The threshold voltage shifted to a lower end and the current intensity increased under the same injury pulses, emulating the allodynia and hyperalgesia effect of a nociceptor.

Having established a robust polymer‐based memristor system with high cycling endurance, we next test its utility as a nociceptive memristor. Nociceptors, which are free nerve cell endings distributed in our skin, can detect signals from damaged tissue, also known as “pain sensors.”^[^ ^60^ ^]^ There are generally four signature nociceptive characteristics for a nociceptor: threshold triggering, no adaptation, relaxation, and sensitization.^[^ ^16^, ^61^ ^]^ These behaviors are explored and demonstrated by our FK‐800 memristor in **Figure** **3**.

When a biological nociceptor receives an external stimulus that is higher than its threshold value, an action potential is induced and the nociceptor sends “threat” neural signals to our brain that are perceived as pain. Otherwise, the nociceptor is not activated. In short, our body will not feel pain unless it is exposed to a sufficiently noxious stimulus.^[^ ^60^, ^61^ ^]^ In an FK‐800 memristor, the threshold behavior is related to the energy required to form conductive filaments between the electrodes. When a sufficiently large voltage pulse is applied, it induces filament formation that bridges the electrodes, and the device switches from a high resistance state to a low resistance state. The threshold triggering of the action potential in a biological nociceptor can be affected by parameters including the intensity, duration, and the number of stimuli—ideally a nociceptor mimic would also have these properties. In the artificial nociceptor, this threshold behavior is emulated by applying a series of voltage pulses with different amplitudes, width, and numbers to the memristor, as displayed in Figure 3a,b,d. Our device does not switch on until the pulse amplitude reached 4 V with a pulse width of 200 ms (Figure 3a). When an external stimulus with the same intensity, 4 V, is applied, the device is not switched on until the pulse width reaches 200 ms, and the current response also increases from 240 to 260 µA with a longer pulse width of 500 ms (Figure 3b), which emulates the effect of stimulus duration on the pain intensity. Note that for all the electrical tests, a sufficient interval time (3 s) is used between pulses so that the device has enough time to relax back to its resting state. As the triggering of the device is also dependent on the number of voltage pulses applied, a series of training pulses with varying amplitudes (3, 4, and 5 V with a pulse width of 20 ms and pulse interval of 2 ms) is used. As shown in Figure 3d, the device is eventually turned on after a certain number of pulses, and the number of pulses required to turn the device on decreases with a higher pulse amplitude (91 pulses for 3 V, 13 pulses at 4 V, and 0 pulses at 5 V). Analogously to the biological systems, we observe thresholds in both duration and intensity of the stimulus for the conductive filaments to bridge the electrodes and switch the device from the “off” to “on” state.

The second characteristic, “no adaptation” is a phenomenon wherein a nociceptor does not adapt to the same continuous noxious stimuli and will continuously send action potentials under extended stimuli, which can protect us from repetitive noxious stimuli.^[^ ^17^ ^]^ As seen in Figure 3d, once the device is switched on, the current response sustains its level (50, 268, 424 µA at 3, 4, 5 V, respectively) despite the application of additional voltage pulses, which mimicks the “no adaptation” behavior of a biological nociceptor that allows us to continuously feel pain under repeated noxious stimuli.

The third characteristic, “relaxation” is related to the stimuli dissipation process of a nociceptor after the noxious stimulus is removed.^[^ ^16^ ^]^ To emulate this behavior, a voltage pulse (4 V, 200 ms) is applied that is sufficient to turn the device into conductive state, followed by a subsequent small reading pulse (2 V, 200 ms) with different interval times (ranging from 0.1 to 3000 ms), and the corresponding current responses are tracked (Figure S13, Supporting Information). Figure 3c shows the current decay as a function of the interval time, which indicates that the device relaxes back to its high resistance state when the noxious stimulus is removed. The increase in resistance after the removal of the bias is attributed to the dissolution of the conductive Ag filaments in the polymer film to minimize the interfacial energy.^[^ ^30^ ^]^ The shorter the time interval, the higher the reading current, which suggests that the device is still active shortly after the removal of the noxious stimulus and a certain amount of time is required for complete relaxation. In this case, it takes at least 3 s for the device to recover after a stimulus of 4 V with the pulse width of 200 ms. We also perform the same study with a stronger noxious stimulus (5 V, see details in Figure S14, Supporting Information) and find that the relaxation time is longer (30 s) than that of the 4 V (3 s), indicating that it takes a longer time to recover from stronger external stimuli. This relaxation process is similar to the injury response of our body as it will recover to the undamaged state after a traumatic injury as time elapses.

The last characteristic, “sensitization” refers to the reduced threshold (allodynia) and augmented response (hyperalgesia) of the sensory nerve fibers to external stimuli after traumatic nerve injury.^[^ ^16^, ^61^ ^]^ In other words, “allodynia” is a condition in which pain is caused by a stimulus that does not normally provoke pain, as indicated by lower threshold triggering and “hyperalgesia” is a condition in which one experiences a higher level of pain. In our device, higher pain levels are indicated by higher currents (lower resistances). Both allodynia and hyperalgesia may help protect vulnerable or predamaged tissues.

To demonstrate the sensitization behavior, we apply a traumatic injury pulse (7 V or 8.5 V with a pulse width of 200 ms) to the device, followed by a series of pulse trains with different amplitudes (1, 2, 3, 4, and 5 V) to record the corresponding current responses 3 min after the injury, as shown in Figure 3e. The maximum current outputs for each pulse train under the normal state (green) and after different levels of traumatic injuries are plotted on logarithmic and linear scales in Figure 3f,g. The plots show that the threshold voltage is reduced after traumatic injuries, which suggests that this device is more easily triggered after injury, emulating the sensing of pain under a stimulus which does not normally elicit pain (allodynia). Meanwhile, compared to the normal state (green), the output current intensities are significantly increased after traumatic injuries, and the stronger the injury, the higher the current. The output current at 4 V is 240 µA without an injury pulse, and increases to 350 and to 530 µA following injury pulses of 7 and 8.5 V, respectively. These results demonstrate a stronger pain intensity under the same external stimulus after an injury impulse. This enhanced sensitivity to noxious stimuli mimicks the hyperalgesia feature of a nociceptor.

Relaxation also applies to injury responses. Not only the injury levels but also the elapsed time post injury plays an important role in the allodynia and hyperalgesia effects.^[^ ^62^ ^]^ To demonstrate post‐traumatic relaxation, an injury pulse of 7 V with a pulse width of 200 ms is applied to a device, followed by a train of pulses of different amplitudes (1, 2, 3, 4, and 5 V) at different time intervals (500 ms, 30 min, and 5.5 h). As shown in current responses in Figure 3h–j, the shorter the time intervals, the lower the threshold voltage, and the higher the current values, which suggest that a reduced threshold (allodynia) sensitization and exaggerated response (hyperalgesia) to noxious stimuli before the predamaged area recovered. About 5.5 h after the injury pulse (7 V), the current intensity is close to the undamaged state, which indicates the device relaxed back to its original state. This study mimicks the recovery of a nociceptor from traumatic injuries over time.

## 5 An Integrated Artificial Injury Response System to Emulate a Sense of Pain, Sign of Injury, and Healing

**Figure 4:**
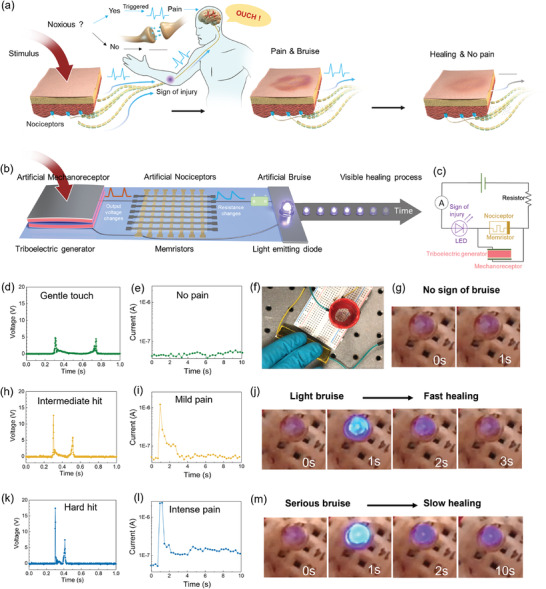
Demonstration of the first bioinspired artificial injury response system with advanced functions, including a sense of pain, sign of injury, and healing, under different scenarios. a) Schematic illustration of the physiological protection modality of human bodies under noxious stimuli, including a sense of pain, sign of injury, and healing. b) Schematic illustration of the bioinspired artificial injury response to emulate a sense of pain, sign of injury and healing based on the integration of a triboelectric generator (artificial mechanoreceptor), a memristor (artificial nociceptor), and a light emitting diode (LED) (artificial bruise) and a power source of 2.4 V. c) Circuit diagram of the corresponding artificial injury response system. d–g) Demonstration of the injury response under a gentle touch. (d) The voltage output generated by a gentle touch on the triboelectric generator, (e) the corresponding memristor circuit current (≈10^−8^A), and (f–g) the photographs of the corresponding state of the LED (off), which suggests a gentle touch would cause no pain nor harm. h–j) Demonstration of the injury response under a mild hit. (h) The voltage output generated by a mild hit on the triboelectric generator. (i) The corresponding memristor circuit current and (j) the photographs of each state of the LED over time, which suggests a mild hit would cause a mild pain and light bruise, but it would heal fast (2 s). k–m) Demonstration of the injury response under a hard hit. (k) The voltage output generated by a hard hit on the triboelectric generator. (l) The corresponding memristor circuit current and (m) the photographs of each state of the LED over time, which suggests a hard hit would cause a more intensive pain (high current) and more serious bruise (higher brightness), and it would take a longer time to recover.

When organisms experience a noxious stimulus, a series of physiological responses are triggered to protect us from harm, including a sense of pain, visible sign of injury, and healing.^[^ ^63^ ^]^ As illustrated in **Figure** **4**a, the nociceptors at the injured site send “threat” signals to our brain that are perceived as pain.^[^ ^60^ ^]^ The damaged capillary endothelium then releases a hormone to narrow the blood vessel to minimize bleeding.^[^ ^64^ ^]^ And the underlying blood clotting protein initiates coagulation and eventually leads to restoration of normal tissue.^[^ ^65^ ^]^ During this process, a visible bruise may change from red to blue over a few hours, then yellow or green after a few days as it heals, and eventually disappears when recovered.^[^ ^65^ ^]^ The unpleasant sensation and visible signs of injury protect organisms from further damage.

To emulate the somatic self‐protective modality under noxious stimuli including a sense of pain, visible signs of injury, and the healing process, we integrate a triboelectric generator (artificial mechanoreceptor), a memristor (artificial nociceptor), a light emitting diode (LED, artificial bruise), and a power supply into a circuit, as shown in Figure 4b. The power supply (2.4 V), a nociceptive memristor, an LED, and an ammeter are connected in series. A triboelectric generator is connected in parallel with the memristor (Figure 4c). In its starting state, the memristor is in a high resistance state (insulating) and thus the LED is off. When a mechanical stimulus is applied to the triboelectric generator, a voltage is generated and applied to the memristor. The output voltage from the triboelectric generator is dependent on the intensity, duration, and frequency of the mechanical stimuli. Once the mechanical stimulus reaches sufficient intensity to generate a voltage that exceeded the memristor threshold, the memristor will switch to a low resistance state (conductive) and the LED will turn on, indicating a sense of pain (high circuit current) and visible sign of bruise (light intensity). With no further action, the memristor relaxes back to its original insulating state; correspondingly, the brightness of the LED then decreases and eventually turns off, indicating the healing process.

Figure 4d–m shows the performance of the artificial injury response system responding to different scenarios where the pain intensity, duration, and frequency were varied.

When a weak mechanical stimulus is applied to the system, the voltage generated from the triboelectric generator does not reach the threshold for the nociceptive memristor to turn on. The memristor remains insulating, and thus the LED remains off and the current in the LED circuit will be low, indicating no damage and no pain (see details in Movie S1, Supporting Information). As demonstrated in Figure 4d–g, with a gentle touch on the triboelectric generator, the output voltage is smaller than the memristor threshold voltage (4 V with a minimum pulse width of 200 ms), the circuit current remains low (≈10^−8^ A), and the LED stays off. These results demonstrate that a gentle touch does not cause damage or pain.

However, as demonstrated in Figure 4h–j, an intermediate impulse to triboelectric generator produces a voltage of ≈13 V, which is sufficiently strong to switch the memristor to a low resistance state (≈10^3^ Ω). The circuit current increases from ≈1 × 10^−8^ to >1 × 10^−6^ A, causing the LED to turn on, indicating a sense of pain and a sign of injury under noxious stimuli. Within 2 s after the noxious stimuli, the circuit current decreases to ≈10^−7^ A and the LED is dimmed; indicative of a healing process, i.e., easing pain and changing bruise coloration. After 3 s, the circuit current drops to <10^−8 ^A and the LED is off, indicating full recovery (see details in Movie S2, Supporting Information).

When a more intense mechanical stimulus is applied to the triboelectric generator, it produces a higher voltage, switching the memristor to an even lower resistance state and resulting in a slower relaxation back to its insulating state. As a result, once turned on, the LED is brighter and it requires longer time to reach the off state, suggestive of a more severe sense of pain and bruising with slower recovery (see details in Movie S3, Supporting Information). As demonstrated in Figure 4k–m, with a hard hit, the triboelectric generator produces a voltage of ≈17 V and set the diffusive memristor to an even lower resistance state (<≈10^3^ Ω). The current immediately increases to 2.5 × 10^−6^ A, rapidly decreases to 1.5 × 10^−7^ A after 2 s and remains almost unchanged for 10 to 60 s (Figure S15 and Movie S3, Supporting Information). Correspondingly, the LED is immediately turned on with a higher brightness compared to that of the intermediate impulse case, and dims slower (>180 s) eventually turning off after hours. This sequence indicates that a stronger noxious stimulus induces a more intense pain with a more serious bruise, and it would take much longer to recover.

## 6 Conclusion

In summary, we develop a new flexible, robust diffusive memristor based on a copolymer of chlorotrifluoroethylene and vinylidene fluoride (FK‐800) that shows outstanding switching endurance of >10^6^ cycles, which is orders of magnitude higher than any other two‐terminal polymer/organic memristors reported in the literature (typically 10^2^–10^3^ cycles) and comparable to that of the state‐of‐art inorganic memristors. The high thermal stability, low thermal expansion coefficient, and high elasticity of FK‐800 is likely contributing to the structural stability under local Joule heating associated with electrical switching, as is verified by in situ conductive AFM studies. With the realization of a flexible robust nociceptive memristor of Ag/FK‐800/Pt, we demonstrate the first bioinspired injury response system with advanced capabilities: a sense of pain, sign of injury, and healing. This artificial self‐protection modality for electronic skin is realized by the effective integration of a triboelectric generator (artificial mechanoreceptor), memristor (artificial nociceptor), and light emitting diode (artificial bruise). Although we demonstrated the nociceptor using just one isolated memristor in this work, we anticipate that arrays of diffusive memristor could be used to identify complex, time‐evolving stimuli. If the stimuli are temporally separated with a time constant that exceeds the relaxation of the memristors, the highly nonlinear IV characteristics of our devices will ensure that only one memristor, corresponding to a unique row‐column combination, responds (assuming opposite polarity applied to rows and columns). However, if distinct stimuli arrive with shorter times, there will be increased probability of sneak‐path formation and generation of a more complicated, time‐dependent response, in addition to the sensitization of specific memristors. While the sneak‐paths could be eliminated by addition of a transistor access devices (the 1T1R) configuration at the cost of increased complexity and power dissipation,^[^ ^66^ ^]^ we anticipate that the emergence of time dependent sneak paths could be used to help identify stimuli in ways that will be presented elsewhere.

On one hand, the material design strategy proposed here may help with future development of flexible robust organic memristors, and the diffusive memristor developed in this work can also be paired with nonvolatile memristor synapse to emulate more advanced neural activities, such as the integrate‐and‐firing properties of neurons. On the other hand, the injury response system demonstrated in this work may inspire the design of new bio‐related applications, e.g., light‐triggered bioresponse systems,^[^ ^67^ ^]^ exemplified by UV‐sensing or UV‐damaging nociceptive systems, or visible light photonic nociceptors with light‐tunable threshold.

## 7 Experimental Section

### Fabrication of FK‐800 Memristors

FK‐800 powder was purchased from 3M (MN, USA). The number‐averaged molecular weight (Mn), weight‐averaged molecular weight (Mw), and glass transition temperature (*T*_g_) were reported as 82 000, 100 000, and 26 °C, respectively. FK‐800 memristors were made with a cross‐bar array structure of Pt/FK‐800/Ag on two substrates; rigid SiO_2_/Si and flexible PET. All substrates were cleaned with ethanol and water and dried with N_2_ gas before deposition of metal electrodes. The bottom electrodes were Ti/Pt with a thickness of 5 nm/100 nm and a feature width of ≈100 µm prepared by e‐beam evaporation with a shadow mask. The thin Ti film served as an adhesion layer to the substrate. Then a thin FK‐800 film (≈200 nm) was spin coated on 25 cm × 25 cm substrate using 40 µL of 3.7 wt% FK‐800 (3M, St. Paul, MN) dissolved in ethyl acetate solution (anhydrous, 99.8%, Sigma‐Aldrich). Spin‐casting was performed using a KW‐4A Spin Coater (Chemat Technology, Inc, CA) with a spin rate of 1500 rpm for 3 s followed by 2000 rpm for 30 s. Afterward, the sample was annealed at 120 °C for 10 min under continuous N_2_ flow, followed by subsequent quenching in a cold‐water bath. This produced a homogeneous amorphous film of FK‐800. The samples were then aged in an oven (BINDER FD 23 Forced Convection Drying and Heating Chamber, Hogentogler & Co. Inc., MD) at 50 °C for 16 h. This caused FK‐800 to crystallize forming micrometer‐scale domains. Finally, the Ag electrode with a thickness of ≈100 nm and line width of 100 µm was deposited on top of the FK‐800 film at right angles to the underlying platinum features by e‐beam evaporation with a shadow mask.

### Fabrication of PVDF Memristors

PVDF samples were prepared for a control study, with the same device geometry, size, and comparable thickness for each layer. The PVDF powder was purchased from Sigma‐Aldrich Co. (MO, USA) and the number‐averaged molecular weight (Mn), weight‐averaged molecular weight (Mw), melting temperature (*T*_m_), and glass transition temperature (*T*_g_) were reported as 71 000, 180 000, 155–165 °C, and −62 °C, respectively. The substrates and cross‐bar electrode arrays used were the same as that of the FK‐800 memristors. For preparation of the PVDF film with a comparable thickness, 40 µL of 12 wt% PVDF in dimethylformamide (DMF) solution (Avantor Performance Materials, Inc, PA) was spin‐casted at a rate of 1200 rpm for 30 s. The sample was then immediately annealed at 90 °C for 10 min using a hot plate.

### Preparation of FK‐800 and PVDF Samples for Conductive AFM Study

For the conductive AFM measurements, a Ag film with the thickness of ≈100 nm was deposited on the SiO_2_/Si substrate by e‐beam evaporation as the bottom electrode. The polymer thin film (either FK‐800 or PVDF) was then spin‐casted on the Ag layer. The c‐AFM tip coated with Pt served as the negative electrode. To improve the chances of finding individual filaments during the in situ electrical switching study, the polymer film was made thinner than that in the bulk device. Specifically, to deposit the FK‐800 thin film onto the substrate, 1 wt% of FK‐800 in ethyl acetate solution was spin‐casted onto the substrate at a rate of 2450 rpm for 3 s and 8000 rpm for 30 s. Then, the sample was annealed at 120 °C for 10 min under N_2_ gas. Afterward, the sample was immediately quenched in cold water bath, and aged at 50 °C for 16 h. To deposit the PVDF thin film with a comparable thickness, 3 wt% of PVDF in DMF solution was spin casted onto the Ag‐coated silicon substrate at 2450 rpm for 3 s and 8000 rpm for 10 s. Then the sample was immediately heated at 90 °C for 10 min on a hot plate. The resulting polymer film thicknesses were ≈20–30 nm.

### Fabrication of a Triboelectric Generator

Fabrication of the triboelectric generator was made from stiff note card paper, graphite, scotch tape, Kapton tape, and 2× common hobbyist stranded electronic jumper wires (schematic in Figure S16, Supporting Information). First, a 3 × 5″ note card was cut into two strips measuring 15 mm × 60 mm and 15 mm × 61 mm. On one side of each of these strips, simple pencil graphite was added to act as an electrode while maintaining a 0.5 mm clean border with the edge of the strip to avoid shorting issues. On the opposite side of one strip scotch tape was adhered and cut to the same dimensions as the strip. The first 10 mm of the jumper wire's sheath were removed to expose the stranded metal wire and were placed in contact with the graphite side of each notecard strip and were taped down with the Kapton tape. Then the two strips with jumper wires attached were taped together, graphite side out edge to edge with Kapton tape. The uneven length of one of the strips creates a gap/arch between the two inner surfaces when tap together. The jumper wire ends were then connected to the breadboard for testing.

### Conductive Atomic Force Microscopy

A Cypher VRS1250 atomic force microscope (Asylum research, Oxford Instruments, Goleta, CA) was utilized to image the samples in this study. All AFM measurements were performed in air at room temperature. Platinum‐coated silicon AFM tip (AC240TM‐R3 cantilevers, Asylum research, Oxford Instruments, Goleta, CA) were used for c‐AFM studies, which also played the role of the negative electrode. The tips had a nominal resonant frequency of 70 kHz, nominal spring constant of 2 N m^−1^, and nominal tip radius of 15 nm. All topography imaging were performed in tapping mode, with typical settings as follows: a free amplitude of 1 V, a set point of 0.8 V, a scan size of 2 µm × 2 µm, a scan rate of 2.44 Hz, and a scanning density of 512 lines per frame. For conductive imaging, contact mode was used with the following settings: free deflection of −0.05 to −0.07 V, set point of 0.000 V, scan size of 2 µm × 2 µm, scan rate of 0.20 Hz, and a scanning density of 64 lines per frame. From the setup, the pixel size and scan rate of the c‐AFM image were determined as 31.7 nm × 31.7 nm and 78 ms per pixel, respectively.

### Electrical Characterizations

All the electrical characterizations were performed in ambient atmosphere. The *IV* sweeps of the memristors were performed with a DC source measuring unit (Keithley 2400) controlled with a Python program. Pulsed measurements were performed using a semiconductor device analyzer (Keysight Technologies, B1500 A) and two waveform generator/fast measurement units (Keysight B1530A) with a probe station (Micromanipulator‐ 450PM‐B).

### Statistical Analysis

All the electrical characterization data was analyzed and plotted via origin. All the AFM images and the corresponding data analysis (RMS roughness) were processed via Gwyddion. For device‐to‐device variability test, ten devices from different batches were tested under the same condition. Standard deviation/average (*σ*/*µ*) of the threshold voltages was used to quantify the sample variations.

## Conflict of Interest

The authors declare no conflict of interest.

## Supporting information

Supporting Information

Supporting Information

Supporting Information

Supporting Information

## Data Availability

The data that support the findings of this study are available from the corresponding author upon reasonable request.
