# Supplementary material for: A Bioinspired Artificial Injury Response System Based on a Robust Polymer Memristor to Mimic a Sense of Pain, Sign of Injury, and Healing
Source: Adv Sci (Weinh). 2022 Mar 25;9(15):2200629. doi: 10.1002/advs.202200629 (PMC9131612; doi:10.1002/advs.202200629)
Supplement: Supplementary file 1 — Supporting Information [file ADVS-9-2200629-s002.pdf]

ADVANCED SCIENCE
Open Access

Supporting Information

for *Adv. Sci.*, DOI 10.1002/advs.202200629

A Bioinspired Artificial Injury Response System Based on a Robust Polymer Memristor to Mimic a Sense of Pain, Sign of Injury, and Healing

Xiaojie Xu, En Ju Cho, Logan Bekker, A. Alec Talin, Elaine Lee, Andrew J. Pascall, Marcus A. Worsley, Jenny Zhou, Caitlyn C. Cook, Joshua D. Kuntz, Seongkoo Cho and Christine A. Orme**

Supporting Information

A Bioinspired Artificial Injury Response System Based on a Robust Polymer Memristor to Mimic a Sense of Pain, Sign of Injury and Healing

Xiaojie Xu, En Ju Cho, Logan Bekker, A. Alec Talin, Elaine Lee, Andrew J. Pascall, Marcus A. Worsley, Jenny Zhou, Caitlyn C. Cook, Joshua D. Kuntz, Seongkoo Cho and Christine A. Orme**

X. Xu, E. Cho, L. Bekker, E. Lee, A. J. Pascall, M. A. Worsley, J. Zhou, C. C. Cook, J. D. Kuntz, S. Cho, C. A. Orme
Lawrence Livermore National Laboratory, 7000 East Avenue, Livermore, CA, 94550, USA
A. A. Talin
Sandia National Laboratories, Livermore, California 94551, USA
E-mail: xu17@llnl.gov; orme1@llnl.gov
Dr. Xu and E. Cho contributed equally to this work.

SI Contents

1. RMS roughness of FK-800 2
2. Threshold Voltage as a Function of Sweep Rate 3
3. Device Variability 4
4. Device Response Time 5
5. Device Performance During Bending 6
6. Endurance of FK-800 Devices 7
7. Endurance of PVDF Devices 8
8. Phase Contrast Images of FK-800 and PVDF Films 9
9. Conductive AFM 10
10. In-situ Conductive AFM Mapping 12
11. Topographic Images of FK-800 Before and After Cycling 13
12. Topographic Images of PVDF Before and After Cycling 14
13. Temporal Relaxation of an FK-800 Device 15
14. Response of Integrated Artificial Injury System 17
15. Triboelectric Generator 18
16. Performance Metrics of Filament-Forming Organic Memristors 20
17. Polymer Properties 21
18. References 22

1. RMS roughness of FK-800

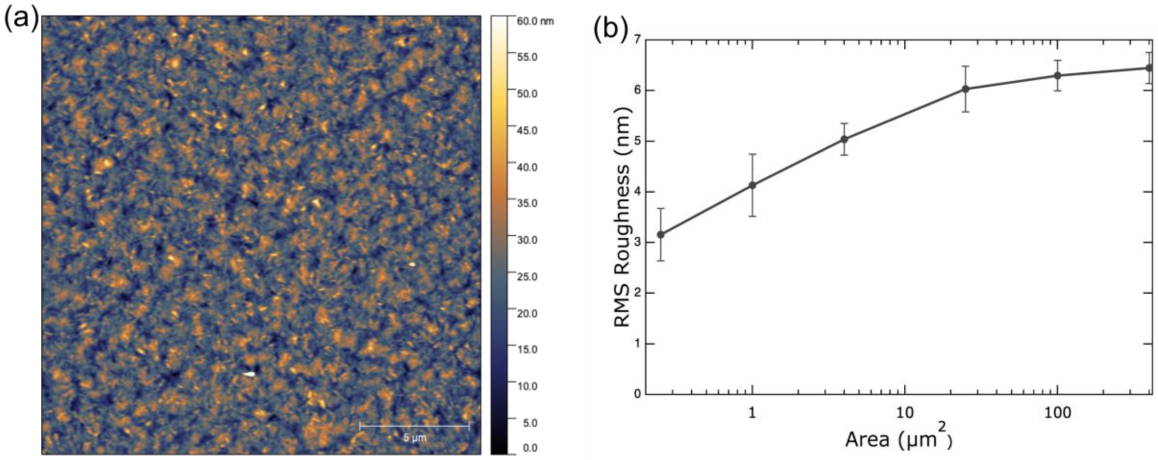

Figure S1. (a) Topographic maps of as-synthesized FK-800 film over 20 µm*20 µm; (b) The root mean square (RMS) roughness of FK-800 as a function of area sizes.

AFM was used to measure FK-800 film topography as a function of image size from 0.5µm to 20µm. Images at a relatively large scale, 20 µm x 20 µm, (Figure S1a) show a uniform distribution of the grains (brighter) and grain boundaries (darker). To ensure that our RMS measurements are representative of the whole, images were taken at 6 locations between cross bars, each more than millimeter apart. Root mean square roughness (Rq) was measured as a function of area (Figure S1b). As expected, roughness increases with area at smaller scales and then levels off when the scale is above the crystal domain size. Our films have an RMS roughness of 4.2nm for 1µmx 1µm, and 6.5nm for 20µm x 20µm images. This RMS value translates to ~3-4% of the thickness at the device scale (100µmx100µm). The surface roughness mainly results from the crystalline grains and grain boundaries. Given conductive filaments are more likely to form at the grain boundaries (verified by Figure 2a-2c), possible approaches to further reduce the cycle-to-cycle and device-to-device variability are to optimize the grain sizes of FK-800 films to guide/confine the filament formation. The grain sizes of FK-800 films can be tuned by adjusting annealing conditions, which will be the focus of our future work.

2. Threshold Voltage as a Function of Sweep Rate

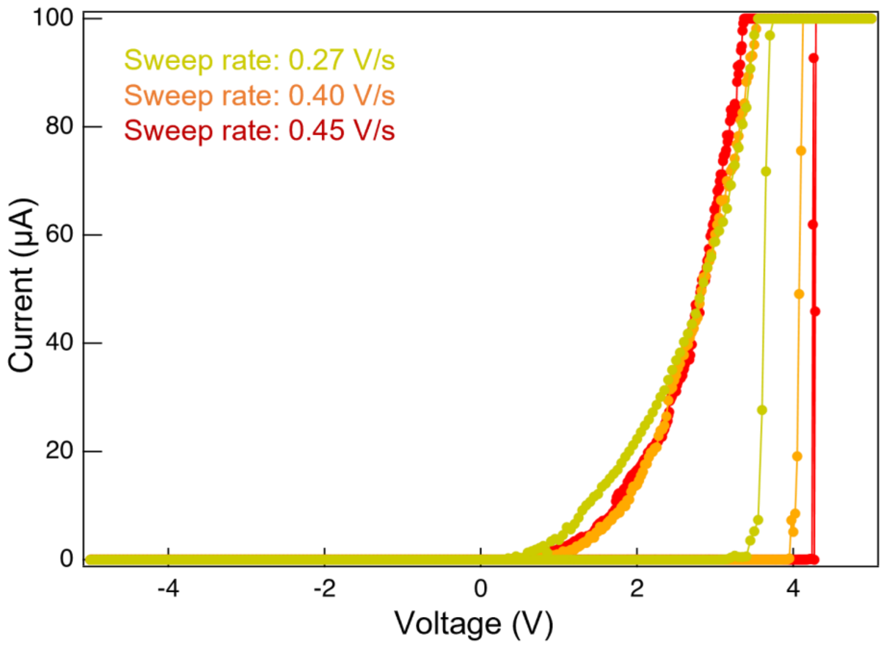

Figure S2. Current-voltage (*I*-*V*) curves of the FK-800 memristor at different sweep rates.

The threshold voltage is not a fixed value due to the dynamic nature of the filament formation.[1] As shown in Figure S2, the threshold voltage of the FK-800 memristor was increased when a faster sweep rate was used. Specifically, the threshold voltage was increased from 3.3 V to 4.4 V when the sweep rate was increased from 0.27 Vs^{-1} to 0.45 Vs^{-1}. These data show that a higher voltage is required to form the Ag filaments that bridge the two electrodes when a faster ramp rate is used, which is consistent with the observations from previous reports. [2,3]

3. Device Variability

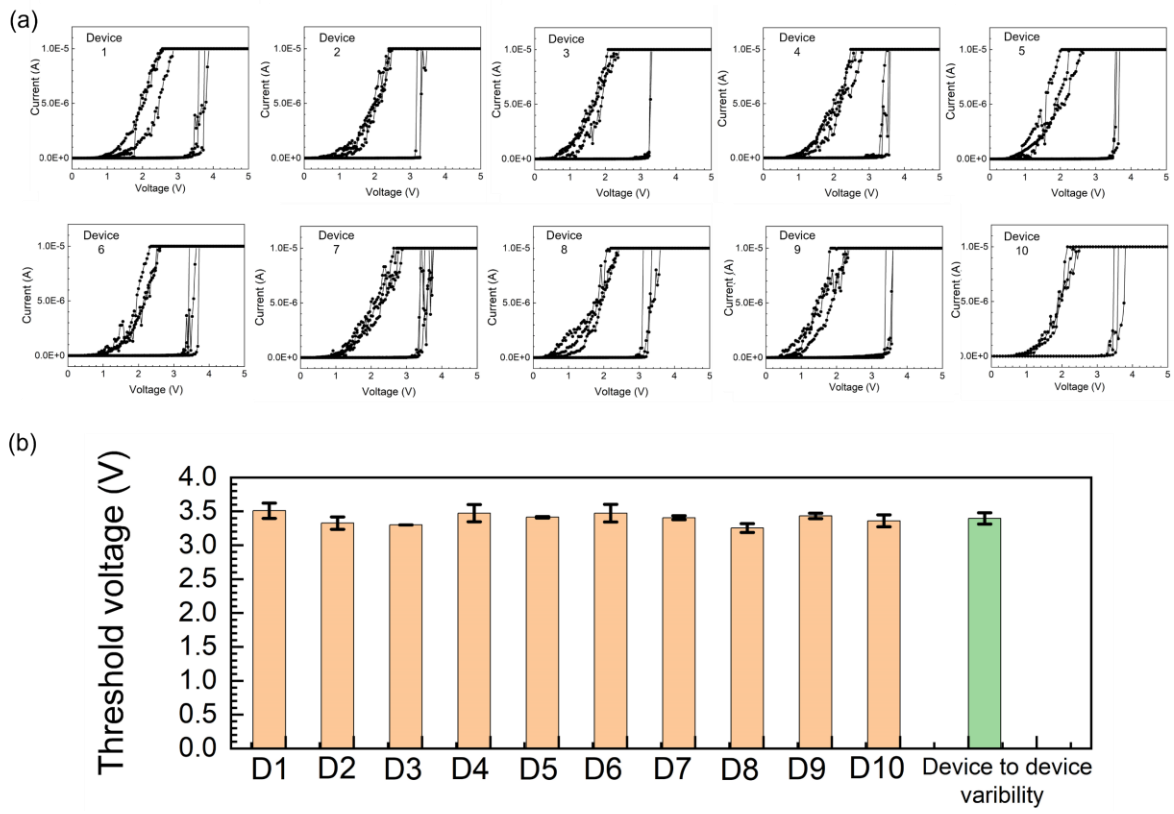

Figure S3. Device to device variability test. (a) Current-voltage (*I*-*V*) sweeps of 10 different FK-800 memristor devices tested under the same conditions; (b) Threshold voltage of 10 different FK-800 memristors. The error bars denote the minimum and maximum values for each category.

To study the device-to-device variability of FK-800 memristors, the *I*-*V* characteristics of 10 different FK-800 memristor devices were tested under the same conditions. The average threshold voltage of the 10 devices was 3.40 ± 0.08 V and the σ/μ (standard deviation/average) was 2.3%, which suggests a low device-to-device variability of FK-800 memristors.

4. Device Response Time

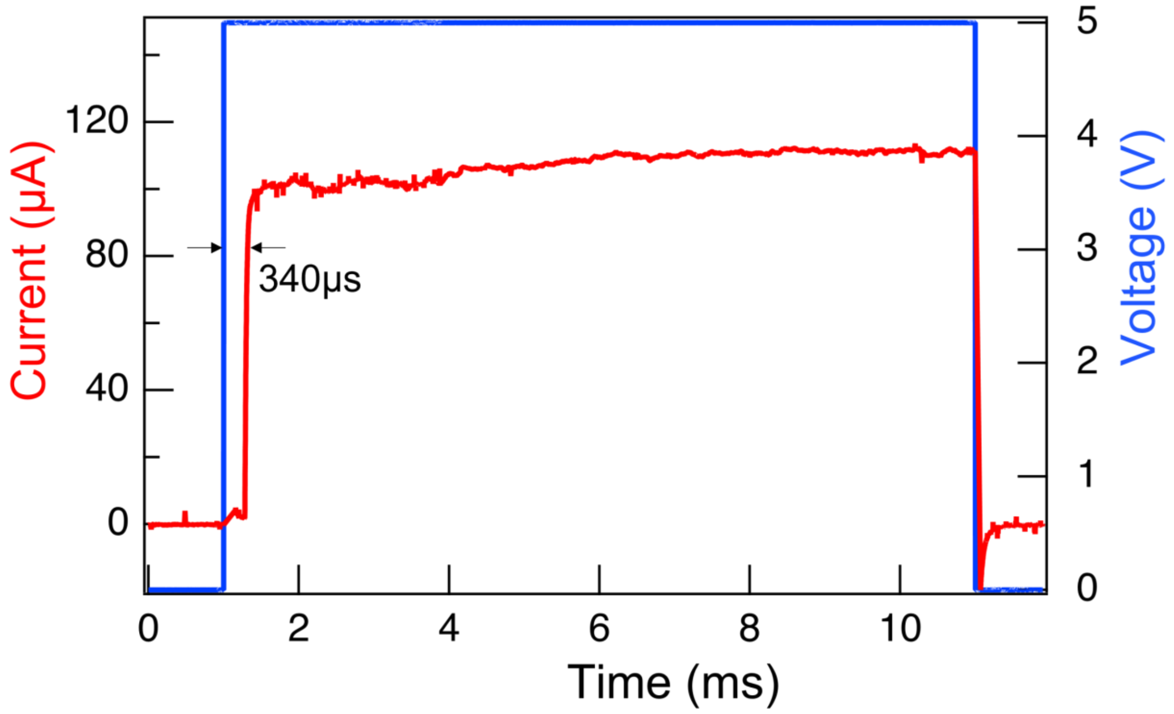

Figure S4. The SET response time of the FK-800 memristor. A testing pulse of 5V with a pulse width of 10 ms and rise/decay time of 1 μs was used.

To study the response time of the FK-800 memristor, a testing pulse of 5V with a pulse width of 10 ms and rise/decay time of 1 μs was used. As shown in Figure S4, the response time of the FK-800 memristor was ~ 340 μs, which is ~30x faster than that of the biological synapses (~ 10 ms). The faster response time suggests the potential of FK-800 memristors to work as artificial synapses with faster processing speeds than the human brain.

5. Device Performance During Bending

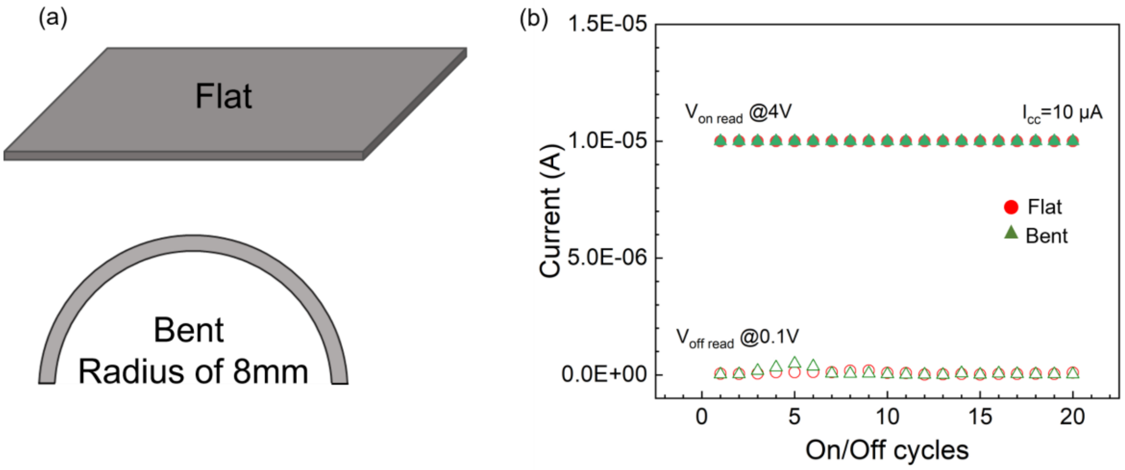

Figure S5. Mechanical flexibility of the FK-800 memristor. (a) Schematic illustration of the device tested under flat and bent condition; (b) Multiple cycles of the on/off switching tests of the device under flat and bent conditions. V_{on} and V_{off} pulses were 4V and 0.1V, respectively, and Icc-compliance current was set to 10 µA to protect the device.

To study the flexibility of the FK-800 memristors, FK-800 memristors were fabricated on a flexible PET substrate. We note that though the same fabrication conditions were used for PET and silicon substrates, the polymer thickness likely differs somewhat due to the difference in wetting behavior of the solvent during spin casting. The on/off switching tests of the device were performed under flat and bent conditions (with a bending radius of 8 mm). The on and off voltages were 4V and 0.1V, respectively. A compliance current (I_{cc}) was set to 10 µA to protect the device. As shown in Figure S5, the device showed stable switching behaviors under flat and bent conditions, which demonstrates its mechanical flexibility and potential for wearable applications.

6. Endurance of FK-800 Devices

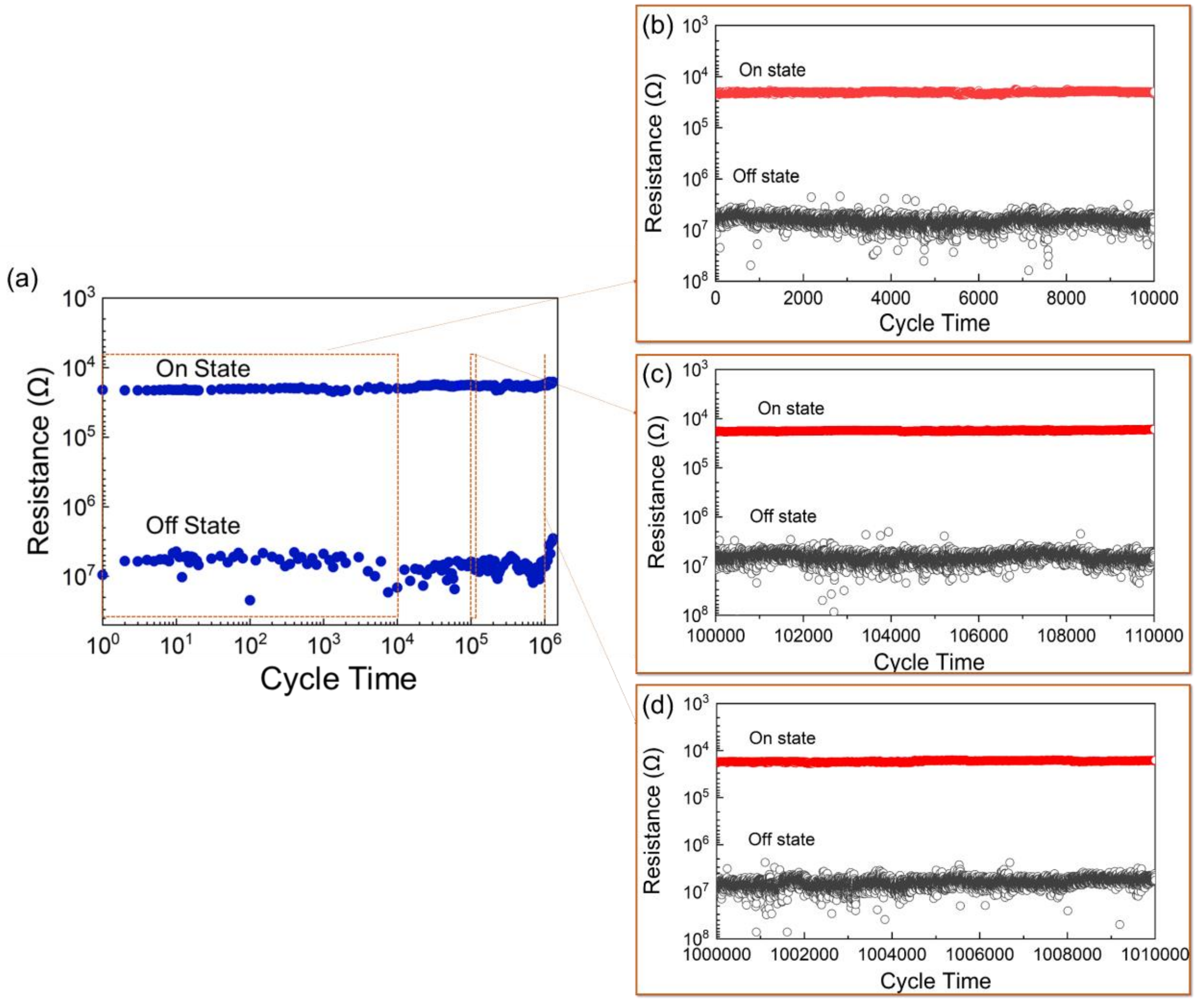

Figure S6. Endurance test of the FK-800 memristor under cycles of electrical switching. The on and off testing pulses were 4.5 V and 0.2 V, with a pulse width of 500 μs and pulse interval of 2 ms.

Figure S6a shows selected data points of the full endurance test (>10^6 cycles). Figure S6b-6c shows the detailed cycle-to-cycle on-off data. For clear identification of individual cycle, Figure S6b-6c displays the enlarged detailed cycle tests of the marked regions (in orange) in Figure S6a. Specifically, Figure S6b displays the first 10k cycles, Figure S6c displays the detailed 10k cycles after 10^5 cycles, and Figure S6d displays the detailed 10k cycles after 10^6 cycles. These plots show that the FK-800 memristor remains almost unchanged even after 10^6 cycles of on-off tests.

7. Endurance of PVDF Devices

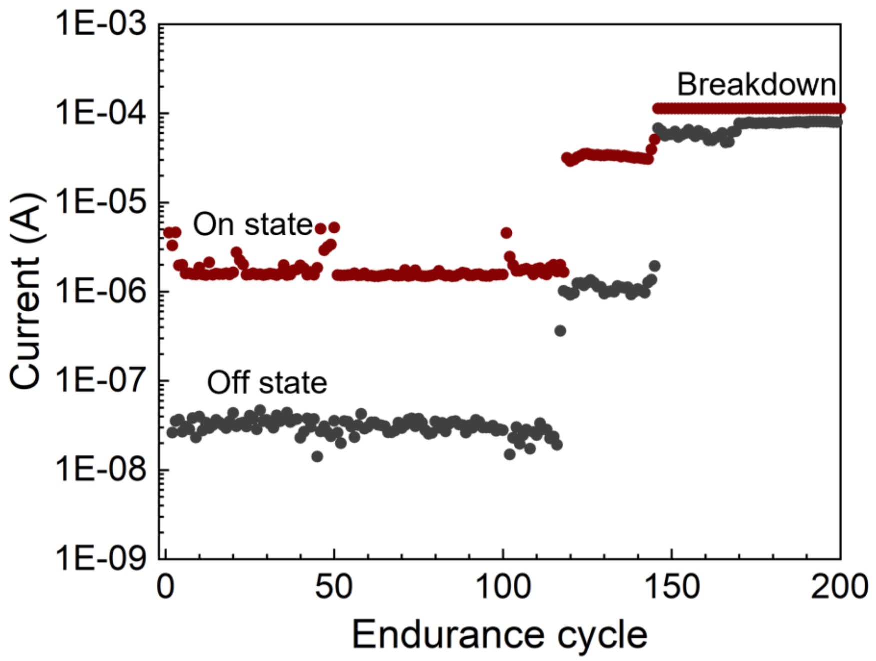

Figure S7. Endurance test of the PVDF memristor under cycles of electrical switching. The on and off testing pulses were 3 V and 0.2 V, respectively, with a pulse width of 500 µs and pulse intervals of 2 ms.

As a control study, PVDF memristors with the same device geometry as those of the FK-800 memristors were made and tested. Figures S7 shows the endurance test of the PVDF memristor under cycles of on/off switching. As can be seen, after 120 cycles of on/off switching, both currents under the high resistance and low resistance were increased and the device was completely broken down after 150 cycles of switching as a result of irreversible shunting. This data suggests that the PVDF memristors have a comparable electrical endurance to that of the other polymer memristors reported in literature (10-10^3 cycles) while the endurance of FK-800 memristors outperformed all other polymer memristors (>10^6 cycles).

8. Phase Contrast Images of FK-800 and PVDF Films

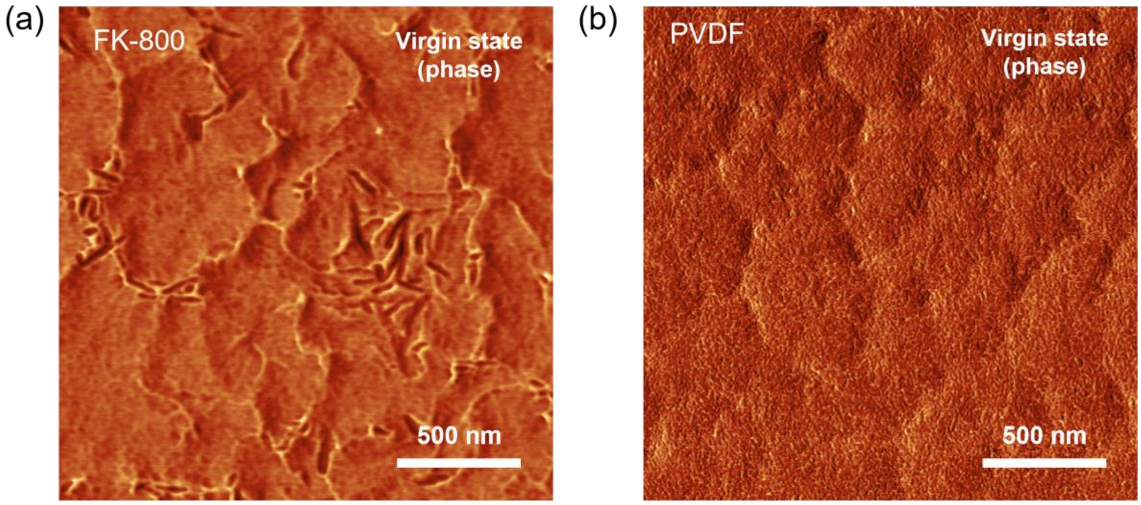

Figure S8. AFM phase images with a scan size of 2µm x 2µm of the (a) FK-800 and (b) PVDF film in the initial virgin state. The phase images were taken using tapping mode. Typical imaging parameters were: free amplitude of 1 V, set point of 0.8 V and scan rate of 2.44 Hz.

To confirm the continuity of the FK-800 and PVDF thin films, AFM phase images were taken of the films in their initial virgin state before a voltage was applied. The color gradient indicates the phase contrast (hardness), where darker coloring indicates harder regions. As polymers are much softer than the Ag and Si substrate, there will be distinctive color contrast if there are pores in the films. As can be seen in Figure S8, there are no significantly dark regions in the films. The images indicate that continuous FK-800 film and PVDF film were coated on the substrates. The slight color contrast at the grain boundaries indicates the different hardness of the crystalline and amorphous regions in the polymer thin films. In particular, dark crystalline plates (oriented perpendicular to the substrate) can be observed in the FK-800 sample.

9. Conductive AFM

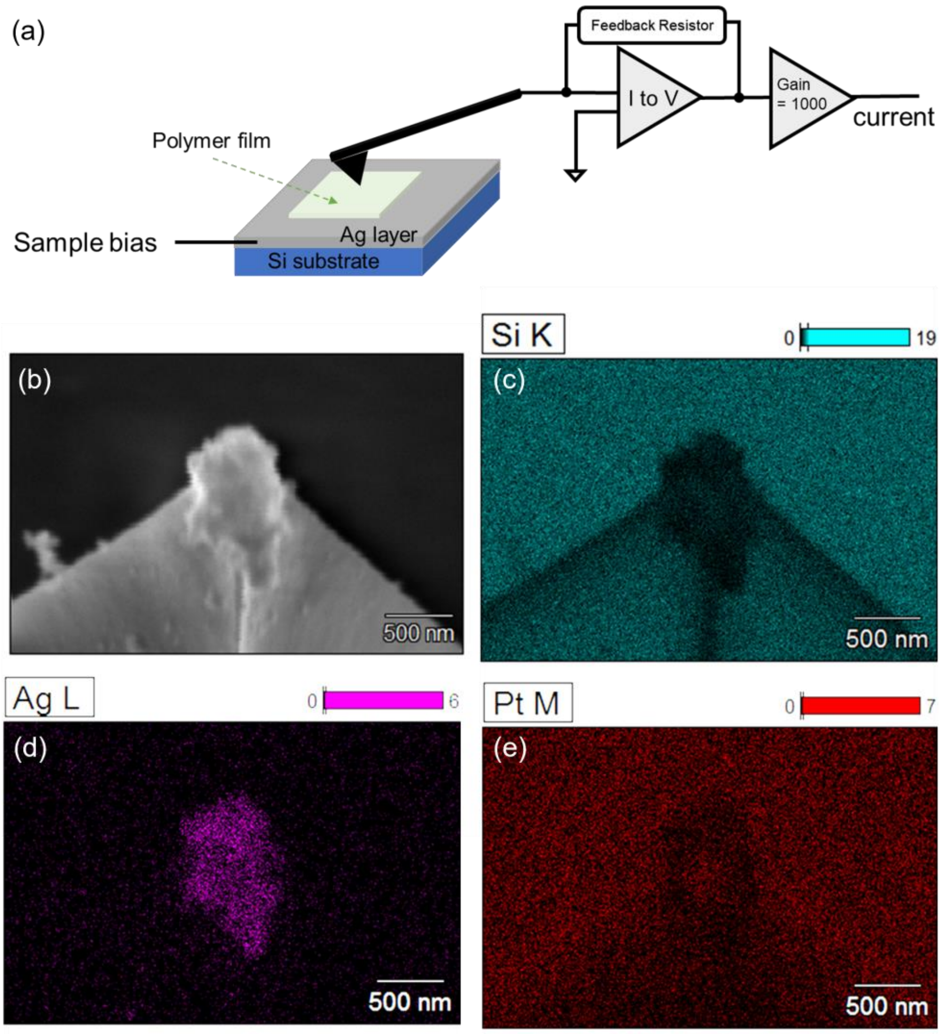

Figure S9. Switching mechanism study of the devices with conductive AFM. (a) Schematic illustration of the experimental set-up for in-situ conductive AFM study. (b) SEM image of the conductive AFM tip after multiple cycles of switching tests. (c)-(e) The corresponding elemental mapping of the tip using Energy Dispersive Spectroscopy (EDS). The element of interest is silicon (Si), silver (Ag) and platinum (Pt), respectively.

To study the switching mechanism of the devices, conductive AFM (c-AFM) was employed to investigate the electrical switching behaviors of the films. As schematically illustrated in Figure S9a, a thin polymer film (~30 nm) was spin cast on a silicon wafer coated with silver (Ag). The Ag film was used the positive electrode, while the c-AFM tip made of silicon and coated with platinum (Pt) was used as the negative electrode. A bias voltage was applied to the silver

electrode on the sample substrate. The c-AFM tip was grounded, so the current would flow across the polymer film between the silver electrode and the AFM tip. Contact mode was used for the c-AFM imaging. A free deflection of -0.05 – -0.07 V, set point of 0.000V, scan size of 2µm x 2µm, scan rate of 0.20Hz, and a scanning density of 64 lines/frame were set for all the study. The pixel size and scan rate of the C-AFM image was determined as 31.7 nm x 31.7 nm and 78ms per pixel, respectively. A bias of 5 V was typically used to turn the device on and 0.2 V to turn the device off.

To verify Ag filaments were the major source for the conductance switching behavior, SEM imaging and Energy Dispersive Spectroscopy (EDS) mapping were performed on a used c-AFM tip (Pt tip) after multiple switching cycles. As seen in Figure S9b, an ~500 nm aggregate was deposited on the c-AFM tip. With elemental mapping, the aggregate was confirmed to be silver (Figure S9d). Maps of the tip materials, Si and Pt, are also shown.

10. In-situ Conductive AFM Mapping

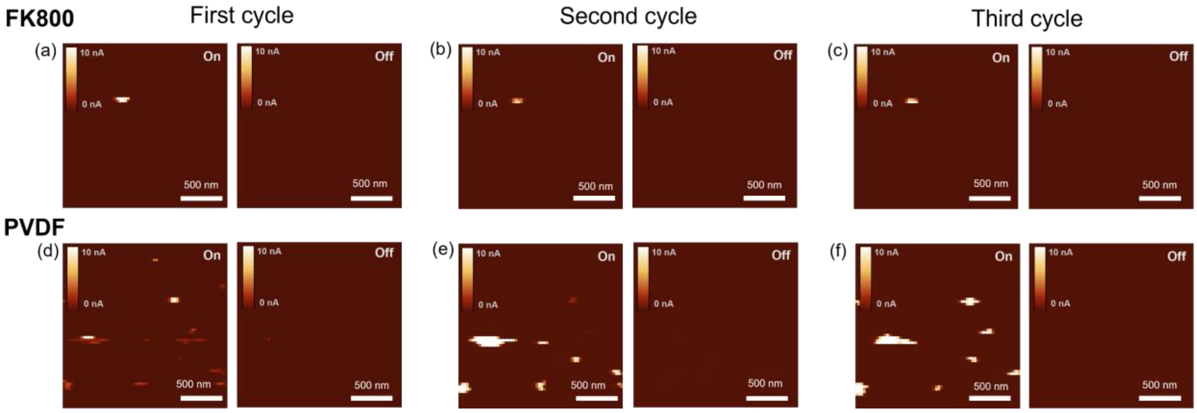

Figure S10. Study of the "On" and "Off" switching cycles of the FK-800 and PVDF devices with conductive AFM.

As shown in Figure S10, current maps after multiple cycles of on (5 V) and off (0.2 V) switching were tracked for the the (a-c) FK-800 and (d-f) PVDF devices. For this study, the c-AFM scan parameters were set as follows: a scan size of 2 x 2µm, rate of 0.20 Hz, scanning density of 64 lines/frame, and current compliance of 10nA was used. The color gradient in the images indicates the current value, with brighter colors indicating higher currents. As seen in Figure S10a, one region (the bright spot) of the FK-800 film was turned on under the bias of 5V and was completely turned off once the bias was reduced to 0.2 V. For the following cycles (Figure S10b and 10c), the same region was consistently turned on and off, which suggests reliable and stable switching at preferential locations. In contrast, a PVDF thin film of comparable thickness were studied under the same condition. As shown in Figure S10d, after applying 5V across the film, multiple regions were turned on with different current densities. For the following cycles (Figure S10e and 10f), the "turned-on" spots were more stochastic. In other words, the active regions didn't consistently align with the previous cycle which suggests that filament formation was stochastic and had higher cycle-to-cycle variability for the PVDF device. In addition, the active spot area also grew after each cycle which we attribute to irreversible morphological changes. These tests demonstrate the more reliable and robust switching characteristics of the FK-800 memristors, which can be further verified by the outstanding endurance (>10^6 cycles) of the FK-800 memristors.

11. Topographic Images of FK-800 Before and After Cycling

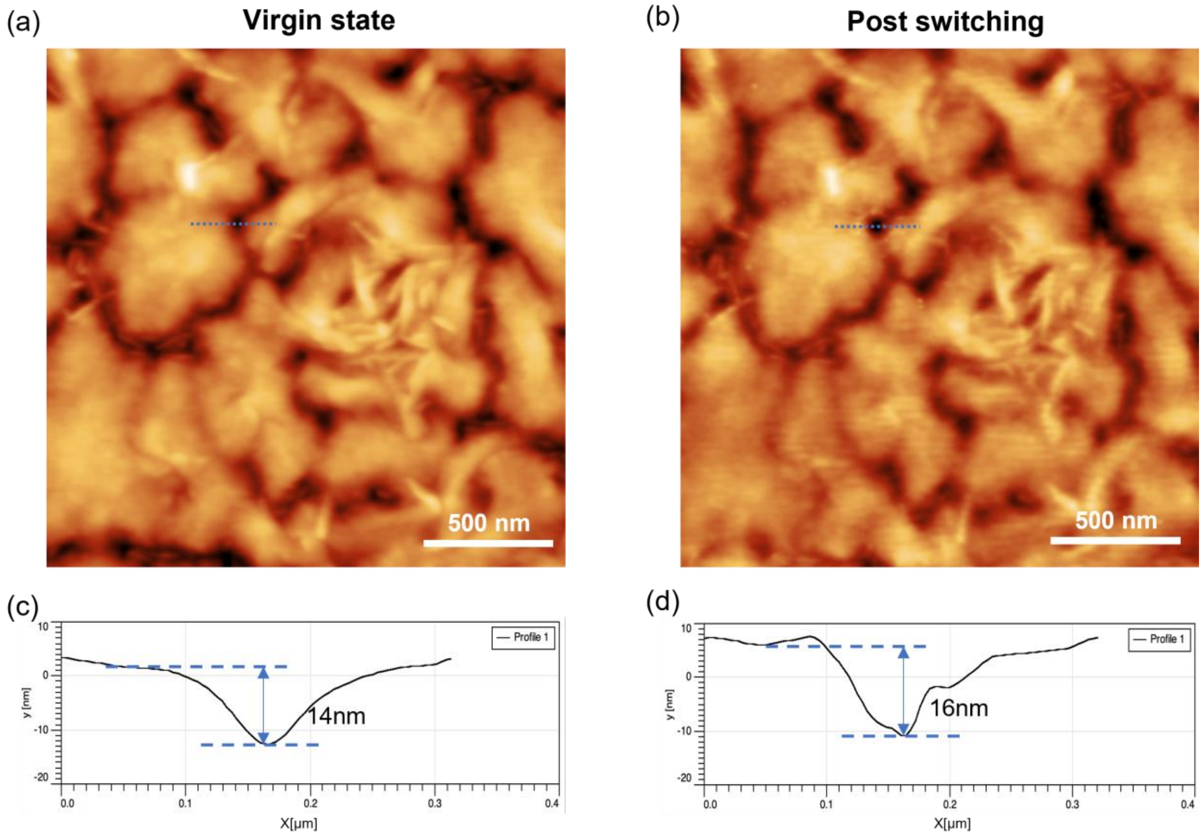

Figure S11. Topography imaging of the FK-800 film at the virgin state and after multiple cycles of on/off switching. The AFM images were scanned under the tapping mode with the typical settings were as follows: a free amplitude of 1V, a set point of 0.8V, a scan size of 2µm x 2µm, a scan rate of 2.44 Hz, and a scanning density of 512 lines/frame.

Figure S11 shows the topography AFM images of the FK-800 film before and after multiple cycles of on/off switching. The dashed line in blue in the images is across the active switching region. The corresponding height trace along the blue line is shown in Figure S11c and S11d. There were no significant topographic changes to the FK-800 film after multiple cycles of switching, which suggests the robustness of the FK-800 memristors.

12. Topographic Images of PVDF Before and After Cycling

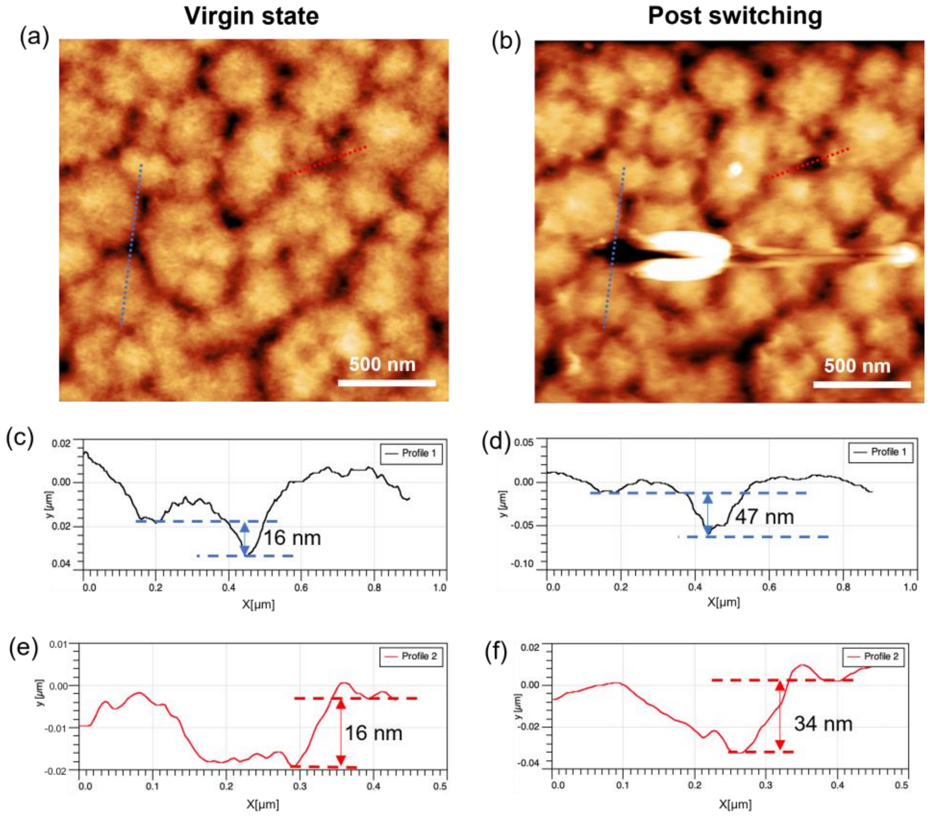

Figure S12. Topography imaging of the PVDF film (a) before and (b) after three cycles of on/off switching. Height profiles across active regions are shown (c,e) before and (d,f) after cycling. The AFM images were scanned using tapping mode with the typical settings were as follows: a free amplitude of 1V, a set point of 0.8V, a scan size of 2µm x 2µm, a scan rate of 2.44 Hz, and a scanning density of 512 lines/frame.

For comparison, a PVDF device with a comparable thickness to that of the FK-800 device was prepared and tested. Figure S12 shows topographic images of the PVDF film before and after three cycles of on/off switching. The dashed line in blue and red in the images are across two active switching regions. The corresponding height trace along these lines are shown in Figure S12c-12f. As can be seen in Figure S12 a and 12b, there were significant morphology differences before and after multiple switching cycles. In particular, material was removed from the active areas resulting in a local deepening at the grain boundary edges. This change in height can be viewed by comparing the height profiles of the blue line before (S12c) and after (S12d) cycling. Similarly, the red line before (S12e) and after (S12f) cycling also showed the local depth increasing by a factor of 2-3 after switching. As local heating due to high current densities

is likely when the film is turned on, the significant morphology changes of the PVDF film after switching suggest that PVDF is not as thermal stabile as FK-800.

13. Temporal Relaxation of an FK-800 Device

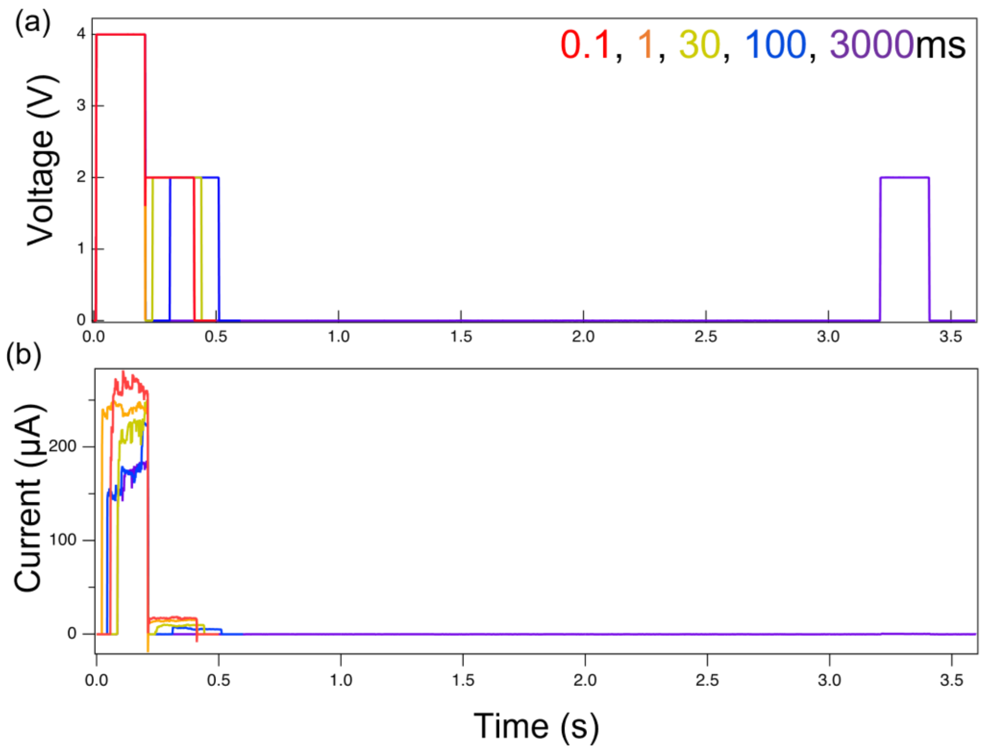

Figure S13. Relaxation behavior of the FK-800 memristor using (a) a 4V voltage pulse (200ms pulse width) followed by a 2 V pulse (200ms pulse width) with different interval times between the two pulses. The designated interval times were 0.1, 1, 30, 100 and 3000ms, respectively. (b) The corresponding currents under the programmed pulses.

As relaxation is one of the signature behaviors of a biological nociceptor. To study relaxation behavior in our devices, a stimulus voltage pulse (4 V) was first applied, followed by a smaller pulse (2 V) at different interval times. The subsequent output currents under the 2 V pulse measured the time-dependent relaxation characteristics. As the interval time increased, the output currents decreased, and eventually turned off (3000 ms), which suggested a transition from an unrelaxed state (<1 ms), to a partially relaxed state (1 ms <t<3000 ms), and eventually to a fully relaxed state (>3000 ms) as time elapsed.

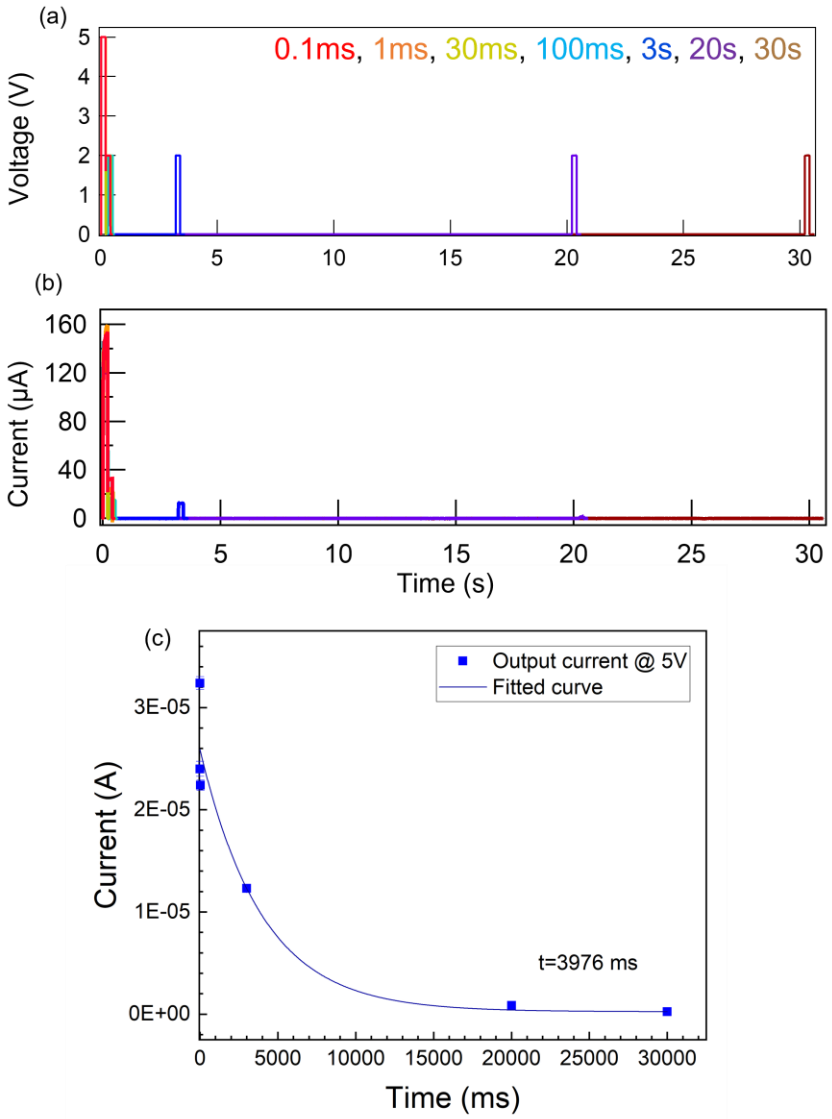

Figure S14. Relaxation behavior of the FK-800 memristor using (a) a 5V voltage pulse (200ms pulse width) followed by a 2 V pulse (200ms pulse width) with different interval times between the two pulses. The designated interval times were 0.1 ms, 1ms, 30ms, 100ms, 3s, 20s and 30s, respectively. (b) The corresponding currents under the programmed pulses. (c) Current outputs at 2V pulse as a function of interval times.

The relaxation behavior of a biological nociceptor is related to the dissipation of the response signals and depends on the intensity of the stimuli. We also investigated the relaxation behavior of the FK-800 memristor using a higher stimulus pulse (5V), followed by the same response pulse (2V) with different interval times. Similar to the observation at 4V in Figure S13, the current showed time-dependent relaxation characteristics. A similar transition from an unrelaxed state, to a partially relaxed state, and eventually to a fully relaxed state as time elapsed

was noticed, except that it took longer for the FK-800 memristor to relax to its original state with a higher stimulus pulse. (Figure S14). Based on the fitting of the current relaxation curve, the decay time constant at a stimulus pulse of 5V is about 3976 ms, which is much longer than that of 4V, which is ~85ms.

14. Response of Integrated Artificial Injury System

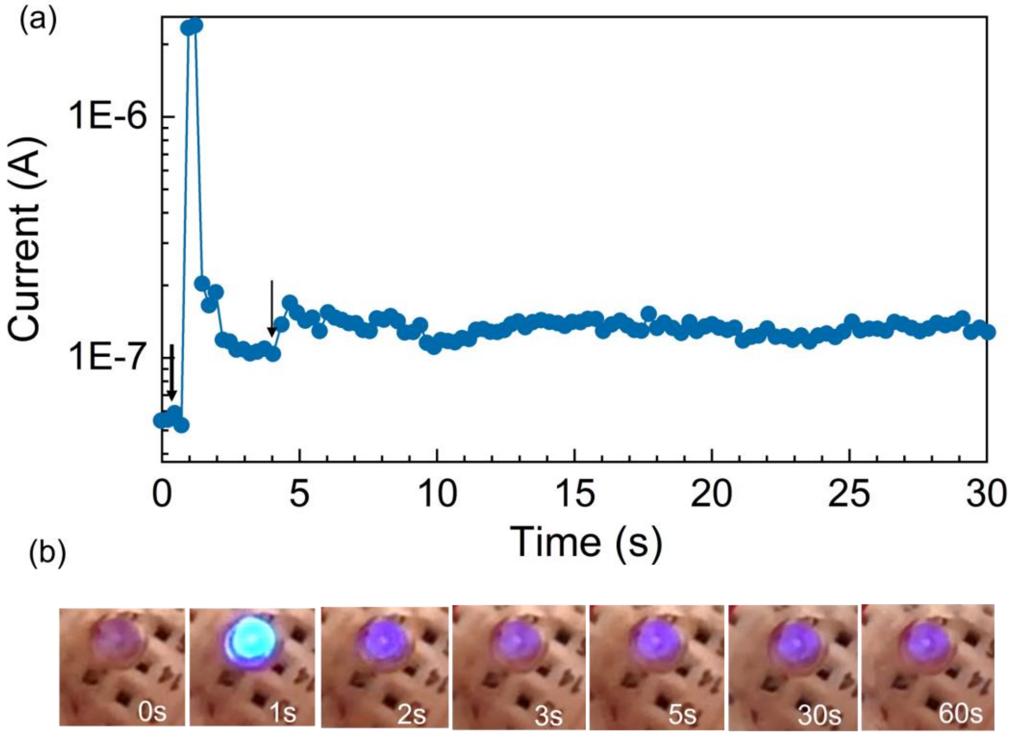

Figure S15. Demonstration of a sense of pain and bruise healing process of the artificial injury response system under a large impulse scenario.

Figure S15 demonstrates the response of the artificial injury response system under a hard injury scenario which includes a high level of pain and a longer healing process. The circuit current which represented the sense of pain (neural signals) and the LED light intensity which represented the sign of injury (artificial bruise) and healing process was simultaneously recorded (Video S3 and Figure S15). As shown in Figure S15a, after a hard hit on the triboelectric generator, the current was immediately increased from 5.5 $x10^{-8}$ A to > 2 $x10^{-6}$ A, which suggested a strong sense of pain was perceived. Simultaneously, the LED was turned on with a high light intensity (See the image at 1s in Figure S15b). Shortly thereafter, the current and LED light intensity decreased, which indicated a reduction in pain and the beginning of

healing. Before the system was fully recovered, another mild hit was applied to the triboelectric generator 4 s after the first hard hit, the current was increased from 1 x10^{-7}A to 1.7 x10^{-7}A and remained almost unchanged after 30 to even 60s, which suggested a longer recovery process was required for a hard injury.

15. Triboelectric Generator

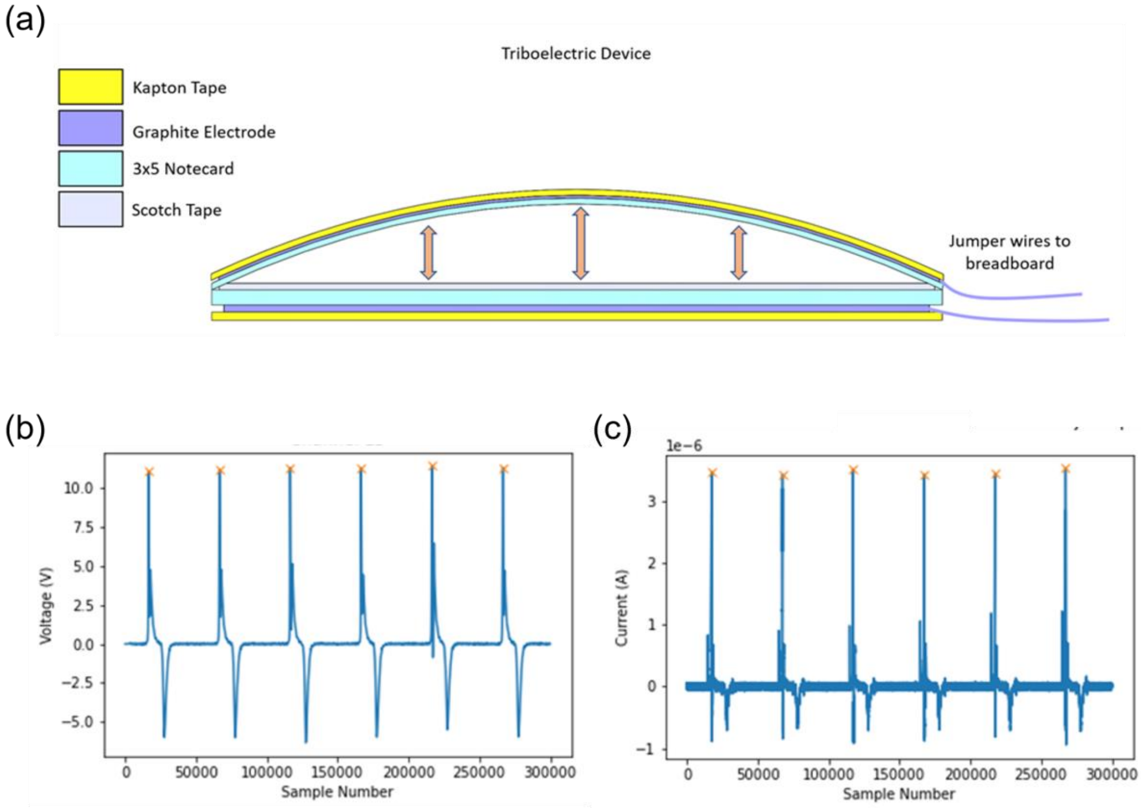

Figure S16. Device architecture and output characteristics of the triboelectric generator fabricated in this work. (a) Schematic illustration of the device architecture of the flexible triboelectric generator; (b) the open circuit voltage (V_{oc}) and (c) short circuit current (I_{sc}) measurements produced by the fabricated triboelectric device under repeated mechanical stimulus.

Triboelectricity is generated through electrostatic induction when two dissimilar materials come into frictional contact with one another.[4] When two materials are brought into contact with one another, surface electrons are able to move from one material to the other (the amount of electron movement is dictated by the materials inherent electron affinity and the difference of that affinity between the two materials). When the two materials are separated, the imbalance

of electrons create a voltage potential between the two surfaces. When both material surfaces are shorted via a wire, the electrons move and equalize the potential, thus driving current.[5,6] The typical current output of a triboelectric material pair appears as an A/C power source as the electrons move back and forth between the two material pairs. The most common way to generate triboelectricity is through contact separation where a material is pressed into another and then lifted normal to their respective surfaces to create a voltage potential.[6,7] Two wires affixed to both material surfaces are connected through a circuit and can then be used to power small electronic devices.

The device used in this experiment is constructed from simple desktop supplies and insulative Kapton tape. The difference in length of the 3x5” notecard strips creates an arch that enables the separation between the inner notecard surface and scotch tape when compressed. A solenoid outputting 5N of force over the 15x60mm (5.5 kPa) triboelectric area at a frequency of 2Hz produced an average open circuit voltage, V_{oc} of 10.3V and a short circuit current, I_{sc} of 3.5µA.

16. Performance Metrics of Filament-Forming Organic Memristors

Table S1. Summary of the memristive behaviors of filament forming organic/polymer memristors reported in literature.

Abbreviations: a) PS:PCBM - Polystyrene: Phenyl-C61-butyric acid methyl ester; b) MABI-

Device structure	Endurance [cycles]	On/Off ratio	Response time [μs]	Set [V]	Set power [W]	Reference
**Ag/FK-800/Pt**	**10^6**	10^3	340	3.5	10^{-4}	**This work**
Ag/DNA:Ag/Pt	100	10^5-10^6	0.02	0.2-0.4	10^{-8}	Angew. Chem. 2020[8]
Ag/carboxymethyl ι-carrageenan/ITO	>1.5 × 10^5	10^4	/	0.4	10^{-7}	Nanoscale, 2019[9]
Au/PS:PCBM/Al a)	500	10^3	/	3.0~4.0	10^{-7}	Adv. Funct. Mater. 2018 [10]
Au/MABI/ITO b)	300	10^2	/	1.50	10^{-4}	Nanoscale, 2018[11]
Al/PVK/ITO c)	/	10^3	/	1.0	10^{-4}	Adv. Mater. 2017[12]
Ag/WK@AuNCs–SF/ITO	100	10^2	/	0.4	10^{-5}	Small 2017[13]
Au/lignin/ITO/PET	/	/	/	0.5~0.7	10^{-4}	ACS Nano 2017[14]
Au/SN:PMMA/p++ Si d)	/	10^6	/	5.0	10^{-6}	ACS Appl. Mater. Interfaces 2017[15]
Ag/sericin/Au e)	21	10^6-10^9	/	2.5	10^{-7}	Adv. Mater. 2013[16]
Au/Co(III) polymer/Au	/	10^2	/	-5.0	/	J. Am. Chem. Soc. 2011 [17]
Ag/PI/GO:PI/PI/ITO f)	130	10^3	/	2.5~4.5	10^{-7}	Appl. Phys. Lett. 2011[18]
Au/PI:PCBM/Al	300	10^3	/	3.0	10^{-5}	Adv. Mater. 2010[19]

perovskite $(CH_3NH_3)_3Bi_2I_9$; c) PVK- Polyvinylcarbazole; d) PMMA - Poly (methyl methacrylate); e) Sericin - Silk protein; f) PI - Polyimide.

17. Polymer Properties

Table S2. Summary of the physical properties of the polymers which have been explored for polymer memristors in literature.

**Polymer**	**PVA**[20]	**PMMA**[21]	**PS**[22]	**PI**[20]	**PVDF**[24]	**FK-800**[25]
**Full name**	Polyvinyl alcohol	Poly (methyl methacrylate)	Polystyrene	Polyimide	Polyvinylidene fluoride	Copolymer of chlorotrifluoroethylene & vinylidene fluoride
**Tg [°C]**	80	105	100	250-340	<0	28-36
**Tm [°C]**	200	160	270	NA	177	105
**Coefficient of thermal expansion at 293K [μm $m^{-1}K^{-1}$]**	70	70-77	70±10	81	120-140	**55±10**
**Thermal degradation temperature [°C]**	200-250	290	270	500	350	**400**
**Young's modulus [GPa]**	0.71	2.855	3.25	4	2.45	**0.16**

18. References

[1] P. Sheridan, K.-H. Kim, S. Gaba, T. Chang, L. Chen, W. Lu, *Nanoscale* **2011**, *3*, 3833.

[2] P. Bousoulas, I. Giannopoulos, P. Asenov, I. Karageorgiou, D. Tsoukalas, *J. Appl. Phys.* **2017**, *121*, 094501.

[3] F. Messerschmitt, M. Kubicek, S. Schweiger, J. L. M. Rupp, *Adv. Funct. Mater.* **2014**, *24*, 7448.

[4] L. Zhou, D. Liu, J. Wang, Z. L. Wang, *Friction* **2020**, *8*, 481.

[5] Y. Wang, Y. Yang, Z. L. Wang, *Npj Flex. Electron.* **2017**, *1*, 1.

[6] W.-G. Kim, D.-W. Kim, I.-W. Tcho, J.-K. Kim, M.-S. Kim, Y.-K. Choi, *ACS Nano* **2021**, *15*, 258.

[7] C. Wu, A. C. Wang, W. Ding, H. Guo, Z. L. Wang, *Adv. Energy Mater.* **2019**, *9*, 1802906.

[8] X. Xu, X. Zhou, T. Wang, X. Shi, Y. Liu, Y. Zuo, L. Xu, M. Wang, X. Hu, X. Yang, J. Chen, X. Yang, L. Chen, P. Chen, H. Peng, *Angew. Chem. Int. Ed.* **2020**, *59*, 12762.

[9] J. Ge, S. Zhang, Z. Liu, Z. Xie, S. Pan, *Nanoscale* **2019**, *11*, 6591.

[10] W. Lee, Y. Kim, Y. Song, K. Cho, D. Yoo, H. Ahn, K. Kang, T. Lee, *Adv. Funct. Mater.* **2018**, *28*, 1801162.

[11] B. Hwang, J.-S. Lee, *Nanoscale* **2018**, *10*, 8578.

[12] H. Ling, M. Yi, M. Nagai, L. Xie, L. Wang, B. Hu, W. Huang, *Adv. Mater.* **2017**, *29*, 1701333.

[13] Y. Xing, C. Shi, J. Zhao, W. Qiu, N. Lin, J. Wang, X. B. Yan, W. D. Yu, X. Y. Liu, *Small* **2017**, *13*, 1702390.

[14] Y. Park, J.-S. Lee, *ACS Nano* **2017**, *11*, 8962.

[15] A.-N. Cha, S.-A. Lee, S. Bae, S. H. Lee, D. S. Lee, G. Wang, T.-W. Kim, *ACS Appl. Mater. Interfaces* **2017**, *9*, 2730.

[16] H. Wang, F. Meng, Y. Cai, L. Zheng, Y. Li, Y. Liu, Y. Jiang, X. Wang, X. Chen, *Adv. Mater.* **2013**, *25*, 5498.

[17] A. Bandyopadhyay, S. Sahu, M. Higuchi, *J. Am. Chem. Soc.* **2011**, *133*, 1168.

[18] C. Wu, F. Li, Y. Zhang, T. Guo, T. Chen, *Appl. Phys. Lett.* **2011**, *99*, 042108.

[19] B. Cho, T.-W. Kim, S. Song, Y. Ji, M. Jo, H. Hwang, G.-Y. Jung, T. Lee, *Adv. Mater.* **2010**, *22*, 1228.

[20] T. S. Gaaz, A. B. Sulong, M. N. Akhtar, A. A. H. Kadhum, A. B. Mohamad, A. A. Al-Amiery, *Molecules* **2015**, *20*, 22833.

[21] N. Patra, A. C. Barone, M. Salerno, *Adv. Polym. Technol.* **2011**, *30*, 12.

[22] F. E. Karasz, H. E. Bair, J. M. O'Reilly, *J. Phys. Chem.* **1965**, *69*, 2657.

[23] S. T. Chen, *MRS Online Proc. Libr.* **1995**, *381*, 141.

[24] G. Laroche, Y. Marois, R. Guidoin, M. W. King, L. Martin, T. How, Y. Douville, *J. Biomed. Mater. Res.* **1995**, *29*, 1525.

[25] W. Cady, L. Caley, *Properties of Kel F-800 Polymer*, **1977**.
